# Cold‐Responsive Hyaluronated Upconversion Nanoplatform for Transdermal Cryo‐Photodynamic Cancer Therapy

**DOI:** 10.1002/advs.202306684

**Published:** 2024-03-14

**Authors:** Anara Molkenova, Hye Eun Choi, Gibum Lee, Hayeon Baek, Mina Kwon, Su Bin Lee, Jeong‐Min Park, Jae‐Hyuk Kim, Dong‐Wook Han, Jungwon Park, Sei Kwang Hahn, Ki Su Kim

**Affiliations:** ^1^ School of Chemical Engineering Department of Organic Materials Science and Engineering Institute for Advanced Organic Materials Pusan National University Busan 46241 Republic of Korea; ^2^ Department of Materials Science and Engineering Pohang University of Science and Technology (POSTECH) Pohang 37673 Republic of Korea; ^3^ School of Chemical and Biological Engineering College of Engineering Seoul National University Seoul 08826 Republic of Korea; ^4^ Department of Civil and Environmental Engineering Pusan National University Busan 46241 Republic of Korea; ^5^ Department of Cogno‐Mechatronics Engineering BIO‐IT Fusion Technology Research Institute Pusan National University Busan 46241 Republic of Korea

**Keywords:** cryotherapy, hyaluronate, photodynamic therapy, synergistic cancer therapy, upconversion nanoparticles

## Abstract

Cryotherapy leverages controlled freezing temperature interventions to engender a cascade of tumor‐suppressing effects. However, its bottleneck lies in the standalone ineffectiveness. A promising strategy is using nanoparticle therapeutics to augment the efficacy of cryotherapy. Here, a cold‐responsive nanoplatform composed of upconversion nanoparticles coated with silica – chlorin e6 – hyaluronic acid (UCNPs@SiO_2_‐Ce6‐HA) is designed. This nanoplatform is employed to integrate cryotherapy with photodynamic therapy (PDT) in order to improve skin cancer treatment efficacy in a synergistic manner. The cryotherapy appeared to enhance the upconversion brightness by suppressing the thermal quenching. The low‐temperature treatment afforded a 2.45‐fold enhancement in the luminescence of UCNPs and a 3.15‐fold increase in the photodynamic efficacy of UCNPs@SiO_2_‐Ce6‐HA nanoplatforms. Ex vivo tests with porcine skins and the subsequent validation in mouse tumor tissues revealed the effective HA‐mediated transdermal delivery of designed nanoplatforms to deep tumor tissues. After transdermal delivery, in vivo photodynamic therapy using the UCNPs@SiO_2_‐Ce6‐HA nanoplatforms resulted in the optimized efficacy of 79% in combination with cryotherapy. These findings underscore the Cryo‐PDT as a truly promising integrated treatment paradigm and warrant further exploring the synergistic interplay between cryotherapy and PDT with bright upconversion to unlock their full potential in cancer therapy.

## Graphical abstract

Transdermal cryo‐photodynamic therapy (Cryo‐PDT) of cancer is demonstrated by using a cold‐responsive nanoplatform of hyaluronic acid, chlorin e6, and silica‐coated upconversion nanoparticles (UCNPs@SiO_2_‐Ce6‐HA). A cryo‐driven boost of upconversion brightness in the nanoplatform results in three‐times higher singlet oxygen generation and remarkable cancer suppression in melanoma‐bearing mice, confirming the feasibility of Cryo‐PDT as a promising therapeutic strategy.

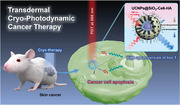

## 1 Introduction

Cryotherapy, including cryosurgery,^[^ ^1^ ^]^ cryoablation,^[^ ^2^ ^]^ and cryospray,^[^ ^3^ ^]^ is a cold‐enabled curative approach to diverse human cancers with lethal repetitive freezing and thawing of cancer cells.^[^ ^4^, ^5^ ^]^ Despite a long history in therapeutic practices, this therapy is insufficient for a complete interception of cancer progression.^[^ ^6^ ^]^ Recently, nanoparticles have been reported to hold great promise for augmenting the efficiency of cancer therapy.^[^ ^7^ ^]^ For example, Wang et al. developed cold‐responsive polymer nanoparticles that release drugs when cooled and generate localized heating under near‐infrared (NIR) laser irradiation, demonstrating the potential for improved breast cancer treatment with cryosurgery.^[^ ^8^ ^]^ Kwak et al. reported the incorporation of thermally conductive inorganic nanoparticles into cryotherapies, such as magnesium oxide, gold, silver, and iron oxide nanoparticles, to maximize the extent of intracellular freezing, drug delivery, and imaging guidance.^[^ ^9^ ^]^ However, their clinical applications remain limited due to the difficulty of intravenously injected nanoparticles to reach the targeted tumor sites, leading to nonspecific biodistribution and unsatisfactory therapeutic effects.^[^ ^10^, ^11^ ^]^ Accordingly, it is still very important to explore the potency of cold‐responsive nanomaterials for the integration into cryotherapy and alternative delivery strategies with an optimal dosing scheme.

Photodynamic therapy (PDT) has attracted huge interest in cancer therapy owing to its non‐invasiveness and minimal side effects. For targeting disease lesions like cancer, PDT relies on the combination of light, photosensitizers, and oxygen. Despite the rich diversity of photosensitizers, the poor skin penetration of ultraviolet (UV) and visible (vis) laser beams seriously constrains the therapeutic potential of PDT to superficial tumors.^[^ ^12^, ^13^ ^]^ To this end, deep tissue penetrable NIR‐responsive nanoscale light transducers, such as upconversion nanoparticles (UCNPs), have become a tailorable UV/vis emission source for PDT under the deep skin.^[^ ^14^, ^15^, ^16^, ^17^ ^]^ However, the use of modest skin‐safe NIR laser doses in PDT elicits the susceptibility of UCNPs to luminescence quenching.^[^ ^18^ ^]^ Moreover, upconversion quenching has multiple origin sources, thereby greatly obscuring a straightforward path toward an ultimate solution.^[^ ^19^ ^]^ Over the past decades, strict limits on the composition of lanthanide dopants in upconversion nanoformulations have been imposed to suppress the concentrated quenching.^[^ ^20^, ^21^ ^]^ The core‐shell structure has been proven to be the most effective configuration of UCNPs to passivate surface quenchers arising from the edge‐defect sites responsible for excitation energy leakage.^[^ ^22^, ^23^ ^]^ Conventional synthetic protocols of core‐shell structures often involve multistep, time‐consuming, and laborious processes, wherein core nanoparticles are produced first and then employed for seed‐mediated epitaxial shell growth via dropwise injection of metal oleate precursors or supply of small cubic phase nanocrystals into the reactor system.^[^ ^24^, ^25^, ^26^, ^27^ ^]^

Thermal quenching is also one of the dominant factors for upconversion quenching since elevated temperature promotes phonon activity accelerating non‐radiative decays.^[^ ^28^, ^29^ ^]^ Very recently, new tantalizing insights were uncovered on the luminescence enhancement of UCNPs at cryogenic temperatures by the suppressed thermal quenching.^[^ ^30^ ^]^ In another recent study, a cryogenic environment has been successfully deployed to narrow the gap between simulation‐predicted and experimentally evidenced luminescence in highly doped UCNPs.^[^ ^31^ ^]^ Triggered by the discovery of cold‐responsive upconversion photoluminescence enhancement, we envisioned the use of cryotherapy to unlock the full therapeutic potential of UCNP‐based nanoplatforms. Excitingly, a clinical report involving patients suggested that cryotherapy and intermittent laser irradiation would serve as cryoanesthesia in PDT without compromising photosensitizer bleaching.^[^ ^32^ ^]^

**Figure 1:**
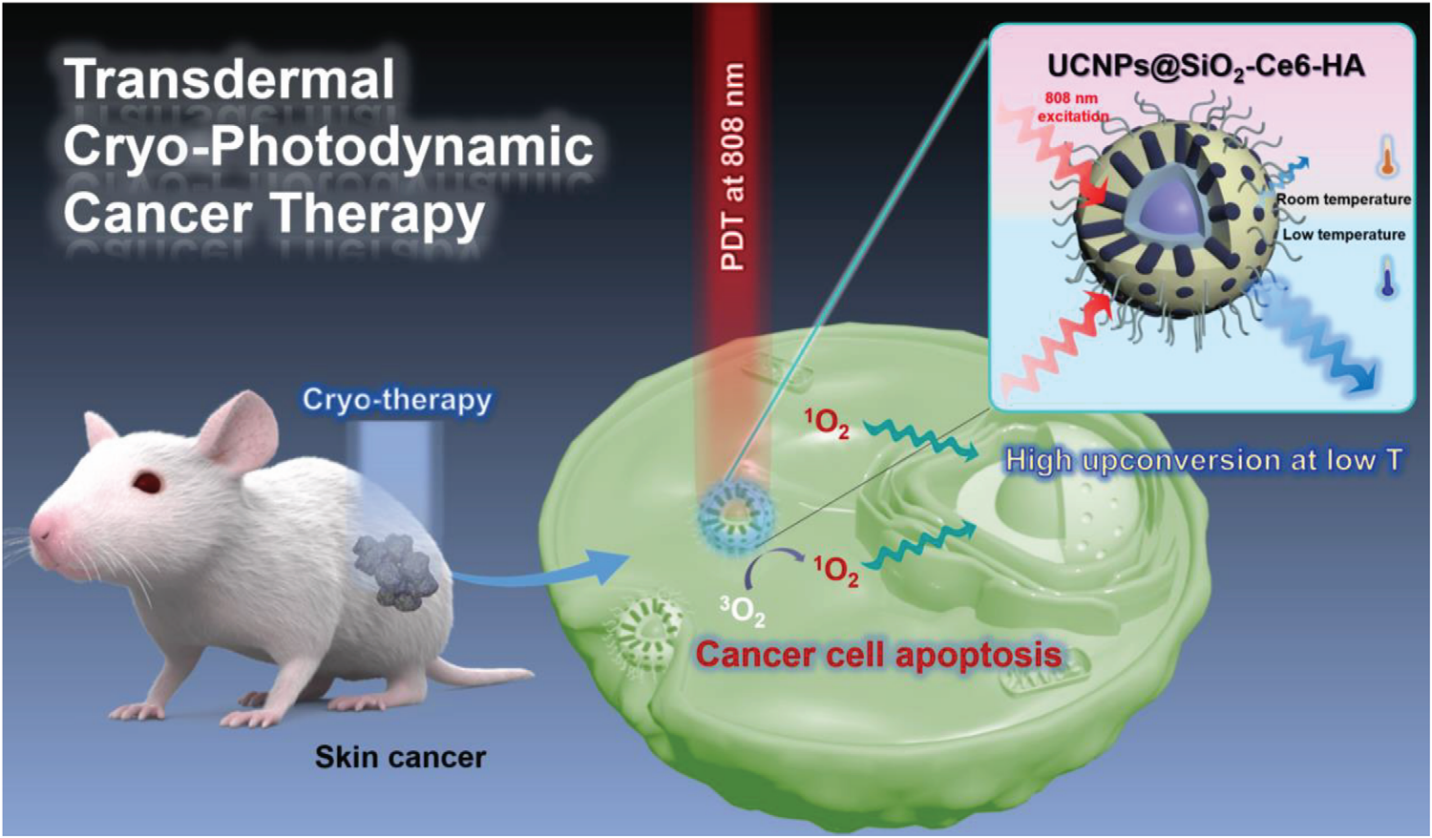
Schematic illustration for the synergistic anticancer efficacy of transdermal Cryo‐PDT treatment against skin melanoma.

Here, we elucidated the high potency of cryotherapy to address the thermal quenching bottleneck of UCNPs with an unprecedented synergistic effect on photodynamic therapy. We prepared 808 nm excitable UCNPs coated with a porous silica shell to load singlet oxygen generating chlorin e6 (Ce6), and then hyaluronic acid (HA) (denoted as UCNPs@SiO_2_‐Ce6‐HA) to realize a more effective cryo‐photodynamic therapy (Cryo‐PDT) of skin melanoma (**Figure** **1**). We particularly focused on designing a facile and accelerated synthetic protocol, which yields highly monodisperse core@shell nanocrystals in a single step via the Ostwald ripening strategy. Unlike the conventional single‐step methods with the injection of metal oleates,^[^ ^27^, ^33^ ^]^ we proposed the continuous injection of pre‐formed precipitate shell precursor solutions upon the synthesis of core nanocrystals for morphological homogeneity via the optimal control of ion composition, reaction time, and pressure in the reactor. The silica shell was employed to stabilize UCNPs in a biological environment and conjugate with Ce6 photosensitizer. The HA‐based surface functionalization facilitated the transdermal delivery of nanoplatforms into the cancerous tissue and further stabilization in a physiological environment.^[^ ^34^, ^35^ ^]^ Moreover, we discovered that cryo‐assisted local temperature tuning could significantly multiply the photoluminescence and Ce6 photosensitization efficiency of UCNPs. We demonstrated that cancer progression could be synergistically suppressed by Cryo‐PDT treatment compared with the individual PDT or cryotherapy treatment. To the best of our knowledge, this is the first report on the use of UCNPs for Cryo‐PDT.

## 2 Results and Discussion

**Figure 2:**
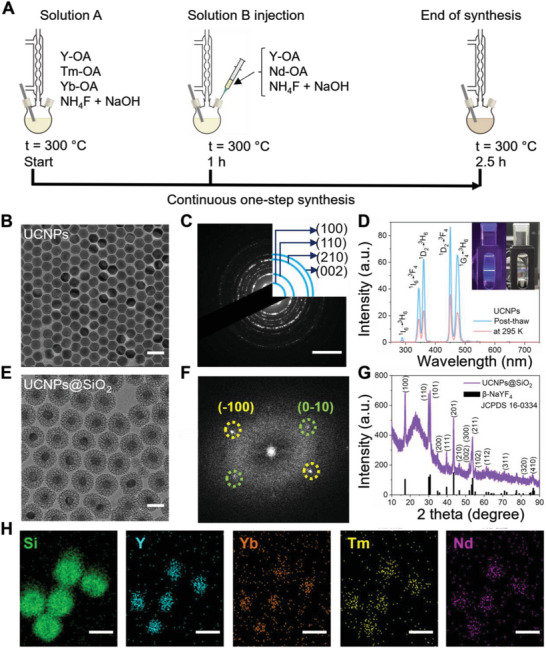
Synthesis and characterization of UCNPs and UCNPs@SiO_2_. A) Schematic illustration for the single‐step synthesis of NaYF_4_:Yb,Tm@NaYF_4_:Nd core@shell UCNPs. B) TEM image of as‐synthesized UCNPs (scale bar: 50 nm). C) SAED pattern of UCNPs (scale bar: 5 nm^−1^). D) Photoluminescence emission spectra of UCNPs solution in cyclohexane post‐thawed and stored at room temperature under 808 nm NIR laser irradiation. Inset: digital images of the naked eye observable blue upconversion emission of post‐thawed UCNPs solution in cyclohexane at room temperature under 808 nm laser irradiation with the light on and off. E) TEM image of silica‐coated NaYF_4_:Yb,Tm@NaYF_4_:Nd nanoparticles (UCNPs@SiO_2_, scale bar: 50 nm). F) FFT from HR‐TEM image of UCNPs@SiO_2_. G) The powder X‐ray diffraction pattern of UCNPs@SiO_2_ sample and the reference pattern of the hexagonal phase β‐NaYF_4_ (JCPDS 16–0334). H) STEM‐EDS elemental mapping of UCNPs@SiO_2_ sample (scale bars: 25 nm).

First, NaYF_4_:Yb,Tm@NaYF_4_:Nd core@shell nanoparticles were prepared in a single step using the Ostwald ripening strategy.^[^ ^36^ ^]^ **Figure** **2**A shows the schematic illustration for the accelerated synthesis pathway in this work. The solution A containing precipitated metal oleates (M‐OA, M = Y, Yb, Tm) was heated to 300 °C to produce monodisperse NaYF_4_:Yb,Tm nanocrystals within 1 h of ripening. Then, the solution B containing pre‐formed precipitate of NaYF_4_:Nd shell precursor was instantly injected at 300 °C into the reactor for continuous synthesis. The advantage of this synthetic design was that the solution B was prepared by mixing metal oleates (M‐OA, M = Y, Nd) with a mixture of freshly made ammonium fluoride (NH_4_F) and sodium hydroxide (NaOH). Such pre‐formed precipitate injection enabled the precise control of sodium fluoride content during the synthesis.^[^ ^37^ ^]^ Notably, because the post‐injection pressure buildup by the residual methanol and moisture was inevitable, we applied a dual nitrogen (N_2_) gas balloon system for our synthesis setup to stabilize the pressure. We also investigated the effect of post‐injection synthesis time on the particle size distribution. The structural characterization revealed that the synthesis for 2.5 h rendered an optimal uniform size, which was validated by the sharp size distribution profile and geometric standard deviation (σ_g_) of 1.046 (Figure S1, Supporting Information). The results revealed that this synthetic condition provided favorable growth kinetics for the final homogeneous core@shell nanocrystals.

Figure 2B shows the transmission electron microscopic (TEM) image of monodisperse NaYF_4_:Yb,Tm@NaYF_4_:Nd core@shell UCNPs with a particle size of ≈32 nm. To validate the formation of hexagonal crystal structure, the selected area electron diffraction (SAED) pattern of a single NaYF_4_:Yb,Tm@NaYF_4_:Nd nanocrystal was analyzed by high resolution‐transmission electron microscopy (HR‐TEM). As shown in Figure 2C, the interplanar d spacings in the ring pattern were measured to be 5.185, 2.999, 1.960, and 1.740 Å, which were well matched with the planes (100), (110), (210), and (002) of hexagonal phase β‐NaYF_4_.^[^ ^38^ ^]^ Furthermore, we used high‐angle annular dark‐field imaging coupled with energy dispersive spectroscopy (HAADF‐EDS) to map the elemental distribution (Figure S2, Supporting Information), which confirmed the presence of Y, Yb, Tm, and Nd elements. As designed, the molar ratio of Y:Yb:Tm:Nd in UCNPs was 81.5:9.0:0.1:9.4 according to the inductively coupled–optical emission spectrometry (ICP‐OES) analysis (Table S1, Supporting Information). All these results confirmed the successful synthesis of NaYF_4_:Yb,Tm@NaYF_4_:Nd core@shell nanoparticles (denoted as UCNPs in the following).

To investigate the effect of temperature on the emission of UCNPs, the photoluminescence (PL) emission spectra of UCNPs in cyclohexane suspension were recorded under 808 nm laser excitation for the identical sample at room temperature (295 K) and post‐thaw conditions (Figure 2D). Post‐thaw was referred to the sample that was kept in the freezer at 193 K for 10 min and then thawed to 295 K before PL measurement. We obtained a 2.45‐fold enhancement of integrated PL emission in the post‐thaw condition, which might be ascribed to its prior exposure to freezing. The exposure to the low‐temperature environment constrained phonon‐induced cross relaxation and maximized the population of the excited states (such as ^1^I_6_, ^1^D_2_, and ^1^G_4_), increasing the radiative emission.^[^ ^31^, ^39^ ^]^ Under NIR laser irradiation, Nd^3+^ ions harvested 808 nm photons to transfer the excitation energy to the ground state of Tm^3+^ ions (^3^H_6_) via Yb^3+^ ions (^2^F_5/2_ → ^2^F_7/2_) by the so‐called cascade energy transfer mechanism depicted in Figure S3 (Supporting Information).^[^ ^40^ ^]^ As a consequence, Tm^3+^ emitter ions produced ultraviolet and visible emissions at 289 nm ^1^I_6_ → ^3^H_6_, 344 nm ^1^I_6_ → ^3^F_4_, 361 nm ^1^D_2_ → ^3^H_6_, 450 nm ^1^D_2_ → ^3^F_4_ and 474 nm ^1^G_4_ → ^3^H_6_.^[^ ^41^ ^]^ Remarkably, the blue upconversion emission of post‐thaw UCNPs could be easily observed by the naked eye under light on and off conditions (Inset of Figure 2D). Thus, there was a clear advantage of cold‐induced modulation of the upconversion brightness to develop a synergistic treatment strategy of cryo‐ and photodynamic therapies.

With the goal of building a therapeutic nanoplatform, UCNPs were coated with a silica shell (UCNPs@SiO_2_) using a Stöber process under the optimized experimental conditions compiled from the previously reported literature protocols.^[^ ^42^, ^43^, ^44^ ^]^ Prior to the synthesis, the hydrophobic surfaces of oleic acid capped UCNPs (UCNPs@OA) were stabilized in water by the bilayer of the cetyltrimethylammonium bromide (CTAB) surfactant. These CTAB‐based surface‐capping also served as a structure‐directing template for the polymerization of silicates to afford a porous silica structure. Under basic conditions (pH 10), cationic (CTAB) and anionic (silicate) species tended to wrap around UCNPs forming a silica–CTAB layer via the electrostatic interaction. The synthesis was conducted under continuous ultrasonic treatment to ensure the isolation of UCNPs prior to encapsulation into a silica layer precluding undesired aggregation (Figure S4, Supporting Information). The successful encapsulation with a porous silica layer was validated by TEM as shown in Figure 2E. The calculated geometric mean diameter of UCNPs@SiO_2_ nanoparticles was 76.214 nm indicating that the average thickness of the silica layer surrounding the UCNP was ≈22 nm (Figure S5, Supporting Information).

Figure 2F shows the fast Fourier transformation (FFT) pattern of the HR‐TEM image taken from a single UCNPs@SiO_2_ nanoparticle for the information on the local diffraction pattern, ensuring the intact hexagonal crystalline structure of UCNPs. The space between the lattice was measured to be 5.112 Å (yellow dotted circle) and 5.192 Å (green dotted circle) in consistent with the hexagonal lattice parameters of NaYF_4_ (5.16552 Å), such as (−1 0 0) and (0 −1 0) planes, respectively. X‐ray diffraction (XRD) pattern of the gross UCNPs@SiO_2_ powder (Figure 2G) showed the presence of the characteristic broad peak of amorphous silica and sharp peaks matching with the reference pattern of pure hexagonal phase β‐NaYF_4_ (JCPDS card 16–0334) without impurity peaks (e.g., cubic phase or NaF). These results confirmed that the hexagonal crystal structure of UCNPs was maintained after the formation of a silica layer under ultrasonic disruptive synthetic conditions. Figure S6 (Supporting Information) shows the HAADF image of UCNPs@SiO_2_ nanoparticles. Notably, the intensity of the HAADF image was proportional to the square of the atomic number of the elements in the sample (I ∝ Z^2^).^[^ ^45^ ^]^ Thus, it could be confirmed that heavy rare earth atoms of UCNPs appeared brighter in the core structure, while lighter atoms like silicon contributed to a weaker intensity of the shell structure. Figure 2H shows the HAADF‐EDS elemental mapping of UCNPs@SiO_2_ nanoparticles to further verify this distribution of the representative elements, such as Y, Yb, Tm, and Nd, in the core structure and Si element predominantly in the outermost shell, ensuring the successful fabrication of UCNPs@SiO_2_. The PL analysis showed a slight decrease in the integrated PL intensity by 22.9% after silica shell encapsulation as shown in Figure S7 (Supporting Information), which could be attributed to the absorbency by the outer silica layer.^[^ ^46^ ^]^ Moreover, we obtained a 2.5‐fold enhancement of integrated PL emission in the UCNPs@SiO_2_ sample exposed at low temperature conditions (Figure S8, Supporting Information). This result indicated that silica shell encapsulation did not weaken the upconversion enhancement effect brought about by low‐temperature treatment.

**Figure 3:**
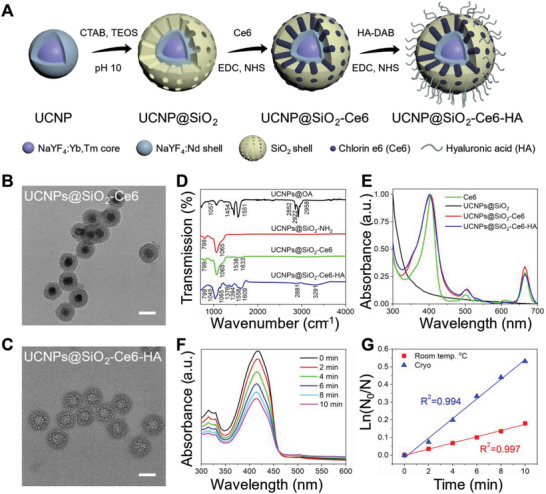
Characteristics of nanoplatforms. A) Schematic illustration for the preparation of UCNPs@SiO_2_‐Ce6‐HA nanoplatforms. TEM images of B) UCNPs@SiO_2_‐Ce6 and C) UCNPs@SiO_2_‐Ce6‐HA (scale bars: 50 nm). D) FT‐IR spectra of UCNPs@OA, UCNPs@SiO_2_‐NH_2_, UCNPs@SiO_2_‐Ce6, and UCNPs@SiO_2_‐Ce6‐HA. E) Normalized absorbance spectra of Ce6, UCNPs@SiO_2_, UCNPs@SiO_2_‐Ce6 and UCNPs@SiO_2_‐Ce6‐HA. F) Singlet oxygen generation profile of UCNPs@SiO_2_‐Ce6‐HA at cryo conditions under 808 nm NIR laser irradiation by using DPBF sensor. G) Comparison of DPBF photodegradation rates by UCNPs@SiO_2_‐Ce6‐HA at cryo (blue) and room temperature (red) conditions.

**Figure** **3**A shows the schematic illustration for the fabrication of UCNPs@SiO_2_‐Ce6‐HA nanoplatforms. Prior to compositing UCNPs@SiO_2_ with Ce6 and HA, it was surface‐modified with 3‐aminopropyltriethoxysilane (APTES) to introduce amine groups, which was confirmed by the zeta potential change from a negative (−25.8 ± 0.84 mV) to a positive value (+38.75 ± 1.04 mV). Then, UCNPs@SiO_2_‐NH_2_ was conjugated with Ce6 via the EDC/NHS chemistry. The loading efficiency of Ce6 in UCNPs@SiO_2_‐Ce6 composite was estimated to be 29.2 wt.%, which was determined by the calibration curve of standard Ce6 solution (Figure S9, Supporting Information). Zeta potential altered to a negative value of −29.84 ± 0.17 mV, reflecting the abundance of carboxylic moieties on the surface of UCNPs@SiO_2_‐Ce6. After that, UCNPs@SiO_2_‐Ce6 was conjugated with HA by the reaction between the carboxyl groups of UCNPs@SiO_2_‐Ce6 and amine groups of HA–diaminobutane (DAB) conjugates. ^1^H NMR analysis showed the characteristic peaks observed at δ 1.9 (HA) and δ 1.6 (DAB) parts per million (ppm) arising from methyl moieties in HA and DAB with the DAB ratio of 32.25% (Figure S10, Supporting Information). TEM images of UCNPs@SiO_2_‐Ce6 and UCNPs@SiO_2_‐Ce6‐HA nanoplatforms also confirmed the successful surface modification of UCNPs@SiO_2_ (Figure 3B,C). Due to the HA coating, the zeta potential was shifted to a slightly negative value of −33.16 ± 0.03 mV, providing its stable colloidal behavior in the physiological environment. Dynamic light scattering (DLS) analysis showed that the hydrodynamic size of UCNPs@SiO_2_‐Ce6‐HA nanoplatforms was ≈177.7 nm with a narrow size distribution (PDI: 0.2448, nearly monodisperse) in phosphate buffered saline (PBS) solution.

FT‐IR spectroscopy was performed to assess the evolution of molecular bonds upon the fabrication of UCNPs@SiO_2_‐Ce6‐HA nanoplatforms (Figure 3D). The peaks at 1065 and 799 cm^−1^ could be assigned to Si‐O‐Si asymmetric and symmetric stretching, respectively, confirming the presence of a silica shell.^[^ ^47^ ^]^ The Ce6 loading was featured by the appearance of new peaks at 1538 cm^−1^ (COO^−^ stretching) and 1633 cm^−1^ (C═N stretching).^[^ ^48^, ^49^ ^]^ The surface modification of UCNPs@SiO_2_‐Ce6 with HA resulted in the peaks of absorption bands at 3291 cm^−1^ (O─H stretching), 2882 cm^−1^ (C─H stretching), 1394 and 1376 cm^−1^ (C─H bending), and 1045 cm^−1^ (C─O stretching).^[^ ^50^ ^]^ The normalized absorbance profiles showed the signal at 410 nm (Figure 3E), which corresponded to the contribution from Ce6, confirming the successful fabrication of UCNPs@SiO_2_‐Ce6‐HA nanoplatforms.

**Figure 4:**
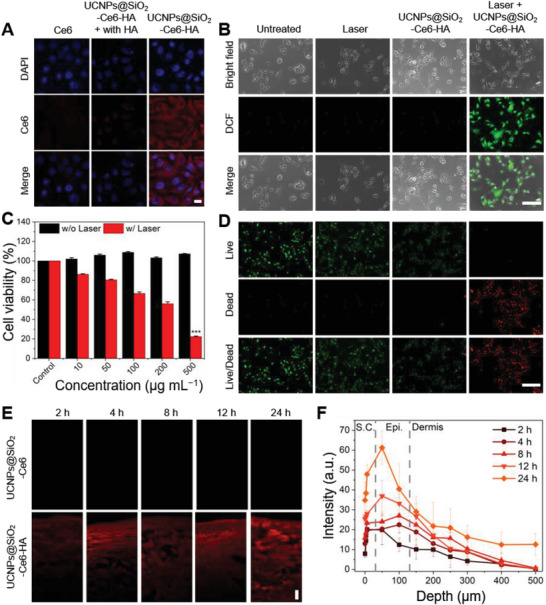
In vitro and ex vivo characteristics of nanoplatforms. A) Confocal microscopy of B16F10 cells after treatment with Ce6 and UCNPs@SiO_2_‐Ce6‐HA with and without HA pre‐incubation (blue: DAPI, red: Ce6, scale bar: 20 µm). B) Intracellular ROS detection in B16F10 cells after various treatments: untreated, 808 nm laser only, and UCNPs@SiO_2_‐Ce6‐HA with or without laser irradiation for 10 min. Green fluorescence from DCF reflects the presence of ROS (scale bar: 150 µm). C) Cell viability of B16F10 cells incubated at various concentrations of UCNPs@SiO_2_‐Ce6‐HA (Control, 10, 50, 100, 200, 500 µg mL^−1^) with (w/) or without (w/o) 808 nm laser irradiation. Error bars represent the standard deviation (SD) from measurements of three (*n* = 3) distinct samples. Unpaired, two‐tailed Student's t‐test was performed to assess statistical significance (^***^ *p* < 0.001). D) Live/dead assay in B16F10 cells after various treatments: untreated (Control), 808 nm laser only (Laser), and UCNPs@SiO_2_‐Ce6‐HA (200 µg mL^−1^) without or with 808 nm laser irradiation for 10 min (green: Calcein AM, red: PI, scale bar: 300 µm). Green or red fluorescence indicates viable or dead cells, respectively. E) Confocal laser scanning micrographs of cryo‐sectioned porcine skin surface after in vitro topical administration of UCNPs@SiO_2_‐Ce6 and UCNPs@SiO_2_‐Ce6‐HA solutions in PBS at a concentration of 500 µg mL^−1^ (scale bar: 50 µm). F) Depth profiles recorded between 2 and 24 h transdermal delivery of UCNPs@SiO_2_‐Ce6‐HA sample into the porcine skin (S.C., stratum corneum; Epi., epidermis).

To get a better understanding on the photochemical events taking place under NIR laser irradiation, the singlet oxygen (^1^O_2_) generation by UCNPs@SiO_2_‐Ce6‐HA nanoplatform was quantified by using probe molecules to detect reactive oxygen species (ROS). The UCNPs of NaYF_4_:Yb,Tm@NaYF_4_:Nd were capable of transducing multiple 808 nm NIR photons energy into high energetic UV photons triggering the photochemical conversion of ^3^O_2_ into cytotoxic ^1^O_2_ by the Ce6 photosensitizer (Figure S11, Supporting Information).^[^ ^51^ ^]^ We used a 1,3‐diphenyl isobenzofuran (DPBF) sensor to capture ^1^O_2_ with its conversion to colorless 1,3‐diphenylisobenzoylbenzene in alcohol/water solution. The ^1^O_2_ generation could be quantitatively estimated from the gradual decrease of its characteristic absorption intensity ≈410 nm on the UV–vis spectra.^[^ ^52^ ^]^ To investigate the temperature‐responsive light‐to‐ROS conversion, we evaluated the DPBF degradation rate under cryo (193 K) and room (295 K) temperature conditions over 10 min, as presented in Figure 3F and Figure S12 (Supporting Information). Strikingly, after excitation with an NIR laser at 808 nm (0.5 W cm^−2^), UCNPs@SiO_2_‐Ce6‐HA nanoplatforms exhibited remarkably higher degradation kinetics at cryo conditions (slope 0.0555, R^2^ = 0.994) as shown in Figure 3G, whereas relatively moderate photooxidation of DPBF was observed at room temperature (slope 0.0176, R^2^ = 0.997). These results confirmed that cryo‐conditions led to a 3.15‐fold enhancement in ^1^O_2_ generation by UCNPs@SiO_2_‐Ce6‐HA nanoplatforms under 10 min NIR laser irradiation. Additional experiments were performed for the UCNPs@SiO_2_‐Ce6 group to assess the effect of HA on the 1,3‐diphenylisobenzofuran (DPBF) photodegradation under both room temperature and cryo conditions (Figure S13, Supporting Information). The DPBF degradation at cryo conditions by UCNPs@SiO_2_‐Ce6 (slope 0.0641, R^2^ = 0.996, Figure S13, Supporting Information) was only 1.15 times higher than that of UCNPs@SiO_2_‐Ce6‐HA (slope 0.0555, R^2^ = 0.994), indicating that the presence of HA coating did not cause the significant ROS generation of the nanoplatform. On the basis of these results, we further conducted in vitro biocompatibility and cellular uptake experiments. Figure S15 (Supporting Information) confirmed UCNPs@SiO_2_‐Ce6‐HA nanoplatforms did not inhibit the growth of mouse fibroblast skin L929 cells and B16F10 melanoma cells even at a high concentration of 500 µg mL^−1^, suggesting the negligible cytotoxicity in the absence of NIR light. Confocal laser scanning microscopy (CLSM) clearly visualized the effective intracellular penetration of UCNPs@SiO_2_‐Ce6‐HA nanoplatforms into B16F10 melanoma cells by HA receptor‐mediated endocytosis (**Figure** **4**A). When HA receptors, such as cluster determinant 44 (CD44) and lymphatic vessel endothelial hyaluronan receptor – 1 (LYVE‐1), were deactivated by pre‐incubation of B16F10 cells with an excessive amount of HA, the cellular uptake of UCNPs@SiO_2_‐Ce6‐HA nanoplatforms were significantly reduced by the competitive binding of HA. As shown in Figure 4A, in the absence of HA, there was a dramatic increase in the red fluorescence intensity from Ce6 around nuclei (blue fluorescence), reflecting the efficient cellular uptake of UCNPs@SiO_2_‐Ce6‐HA nanoplatforms by the HA receptor‐mediated endocytosis. For further validation, using normal and cancer cells, we compared the cellular uptake of Ce6 photosensitizer, UCNPs@SiO_2_‐Ce6 and UCNPs@SiO_2_‐Ce6‐HA with and without excess of HA. As shown in Figure S16A (Supporting Information), there was no significant accumulation of Ce6 and UCNPs@SiO_2_‐Ce6 in either cell type, which was observed through the relatively weak red fluorescence of Ce6. A similar scenario was observed in the dual presence of HA and UCNPs@SiO_2_‐Ce6‐HA, which confirmed that excess HA remarkably suppressed the cellular uptake of the nanoplatform. In consistent with the previous reports on HA receptor mediated endocytosis,^[^ ^53^ ^]^ UCNPs@SiO_2_‐Ce6‐HA displayed a more abundant accumulation in B16F10 cells than in L929 cells. This cancer‐cell specific accumulation was determined by 1.74‐fold increase in the red fluorescence signal intensity (Figure S16B, Supporting Information). Thus, we could confirm the role of HA in the tumor targeted accumulation of UCNPs@SiO_2_‐Ce6‐HA nanoplatform, which attenuated the threat of undesired ″off‐tumor′ toxicity.

In vitro PDT of UCNPs@SiO_2_‐Ce6‐HA nanoplatform was carried out to assess its antitumor therapeutic efficacy. The generation of intracellular ROS was visualized by the singlet oxygen‐triggered conversion of 2,7‐dichlorodihydrofluorescein (DCFH‐DA) into its oxidized DCF with green fluorescence.^[^ ^54^ ^]^ Figure 4B shows the fluorescence microscopic images of the control, laser, and UCNPs@SiO_2_‐Ce6‐HA treated groups with no obvious green fluorescence indicating none or negligible ROS generation. In contrast, clear green fluorescence was observed in B16F10 cells incubated with UCNPs@SiO_2_‐Ce6‐HA followed by 808 nm laser irradiation (Laser + UCNPs@SiO_2_‐Ce6‐HA). The fluorescence intensity was increased 113‐fold compared to the other treatment groups, showing the efficient intracellular ROS generation.

In vitro NIR‐mediated antitumor effect of UCNPs@SiO_2_‐Ce6‐HA nanoplatform was quantitatively investigated by using CCK‐8 assays. B16F10 cells were incubated for 24 h with UCNPs@SiO_2_‐Ce6‐HA nanoplatforms at various concentrations from 0 to 500 µg mL^−1^. After that, cells were irradiated with an 808 nm NIR laser for 10 min at a power density of 0.5 W cm^−2^. As shown in Figure 4C, upon 808 nm NIR illumination, the viability of B16F10 cells dropped to 22.3% at a concentration of 500 µg mL^−1^. To further validate the fore‐going experimental results, in vitro antitumor therapeutic effect was also visualized by live and dead cells co‐staining assay with Calcein AM and propidium iodide (PI) and illustrated in Figure 4D. While the reference treatment with the control, laser, and UCNPs@SiO_2_‐Ce6‐HA nanoplatforms exhibited no obvious toxicity, the treatment with UCNPs@SiO_2_‐Ce6‐HA nanoplatforms plus 808 nm laser exhibited little green fluorescence (live cells) and strong red fluorescence (dead cells). These results confirmed that UCNPs@SiO_2_‐Ce6‐HA nanoplatform showed high biocompatibility and NIR‐mediated anticancer efficacy.

In vitro transdermal delivery of UCNPs@SiO_2_‐Ce6‐HA nanoplatforms was assessed by using porcine skin with the anatomical and physiological similarities to human skin.^[^ ^55^ ^]^ HA is one of the essential biological building blocks of epidermis and dermis in human skin, and known to facilitate the transdermal delivery of drugs deep into the dermis.^[^ ^56^, ^57^, ^58^ ^]^, Figure 4E shows the CLSM images and the depth profiles of cryo‐sectioned porcine skin surface after in vitro topical administration of UCNPs@SiO_2_‐Ce6 and UCNPs@SiO_2_‐Ce6‐HA nanoplatforms solutions in PBS. The extent of dermal penetration was monitored for 24 h by using red fluorescence signal from Ce6 photosensitizers in the skin. Figure 4E and Figure S17 (Supporting Information) show a marked difference in the dermal penetration between UCNPs@SiO_2_‐Ce6 and UCNPs@SiO_2_‐Ce6‐HA nanoparticles. HA coated nanoparticles were evenly distributed with bright red fluorescence in the deep skin layers. The intensity gradually increased over time reflecting the effective transdermal delivery of UCNPs@SiO_2_‐Ce6‐HA nanoplatforms through the stratum corneum and the underlying porcine skin layers. These results were also confirmed by the depth profile analysis as shown in Figure 4F. More specifically, the integrated red fluorescence intensity area increased 3.2‐fold in the epidermis layer between 2 and 24 h. As expected, the diffusion of UCNPs@SiO_2_‐Ce6 was hindered by the skin barrier and mainly accumulated in the stratum corneum. These results were well matched with other reports elsewhere,^[^ ^53^, ^54^ ^]^ which proved the superior potential of UCNPs@SiO_2_‐Ce6‐HA nanoplatforms for the effective transdermal delivery into deep skin layers and the cancerous cells. HA derivatives are also known to facilitate the delivery into tumor cells with abundant HA receptors such as CD44, LYVE‐1, and others.^[^ ^35^ ^]^

Inspired by the remarkable in vitro performance for PDT and transdermal delivery, we further investigated in vivo antitumor effect of UCNPs@SiO_2_‐Ce6‐HA nanoplatform in B16F10 tumor‐bearing C57BL/6 mice. To maximize the PDT efficacy of UCNPs@SiO_2_‐Ce6‐HA nanoplatform, we employed cryotherapy against melanoma tumors. B16F10 tumor‐bearing mice were randomly distributed into seven groups as follows: 1) PBS (Control), 2); UCNPs@SiO_2_‐Ce6‐HA, 3) Cryo, 4) Laser, 5) Cryo + UCNPs@SiO_2_‐Ce6‐HA, 6) Laser + UCNPs@SiO_2_‐Ce6‐HA (PDT), 7) Cryo + Laser + UCNPs@SiO_2_‐Ce6‐HA (Cryo‐PDT).

**Figure 5:**
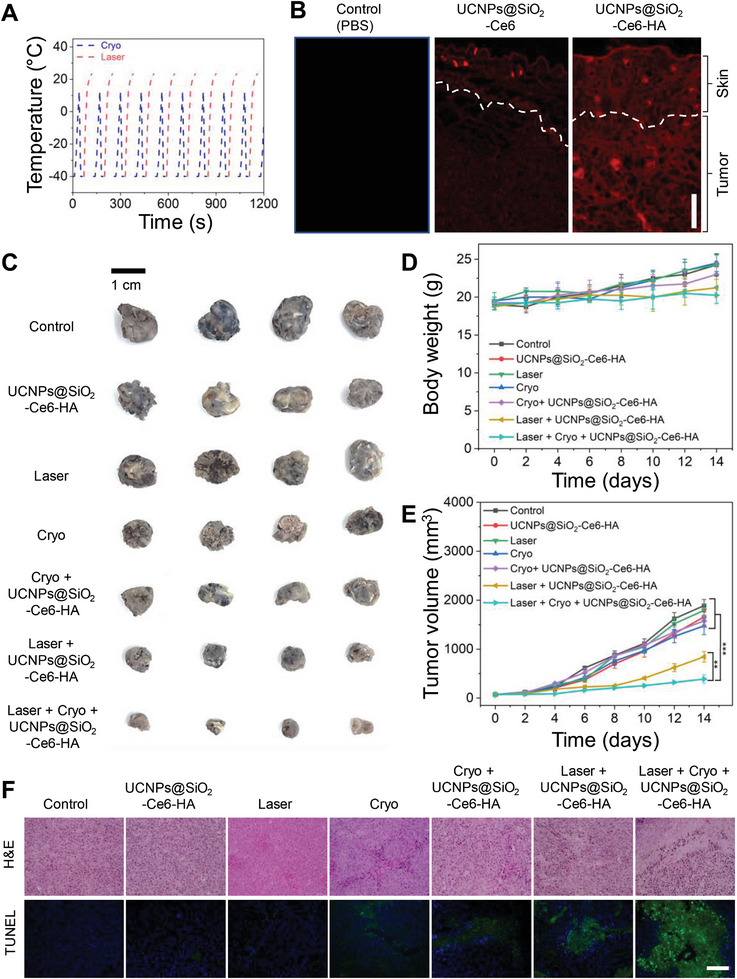
In vivo cryo‐photodynamic therapy. A) Temperature change profiles over Cryo‐PDT treatment cycles, where each cycle includes 1 min Cryo and 1 min Laser (total 10 cycles). According to the FLIR infra‐red camera user manual, its lowest temperature measurement range was limited to −20 °C. B) CLSM images of B16F10 tumor cryo‐sections after in vivo topical administration of UCNPs@SiO_2_‐Ce6 and UCNPs@SiO_2_‐Ce6‐HA solutions in PBS at a concentration of 500 µg mL^−1^ (scale bar: 150 µm). The dashed line indicates the skin/tumor boundary. C) Representative photographs of harvested melanoma tumor tissues after various treatments in C57BL6 mice: 1) Control; 2) UCNPs@SiO_2_‐Ce6‐HA; 3) Cryo; 4) Laser; 5) Cryo + UCNPs@SiO_2_‐Ce6‐HA; 6) Laser + UCNPs@SiO_2_‐Ce6‐HA; and 7) Cryo + Laser + UCNPs@SiO_2_‐Ce6‐HA (scale bar: 1 cm). D) Body weight and E) tumor growth profiles after various treatments for 14 days. Error bars represent the standard deviation (SD) from measurements of four (*n* = 4) distinct samples. One‐way ANOVA with Tukey's post‐hoc test was performed to assess statistical significance (^**^ *p* < 0.01 and ^***^ *p* < 0.001). F) H&E and TUNEL staining of tumor tissues after various treatments (scale bar: 150 µm).

The tumor regions of groups 2, 5, 6, and 7 were treated by the topical administration of UCNPs@SiO_2_‐Ce6‐HA solution (500 µg mL^−1^, 50 µL) in PBS for 30 min. In the Cryo treatment groups 3 and 5, tumor regions exclusively were treated with liquid nitrogen for 10 min minimizing the risk of negative effect on surrounding normal cells. The Laser treatment groups 4 and 6 were exposed to 808 nm NIR laser (0.5 W cm^−2^, 10 min) irradiation. Figure S18 (Supporting Information) shows the schematic illustration for the laser setup. In group 7, the topical administration of UCNPs@SiO_2_‐Ce6‐HA solution (30 min) was followed by the consecutive 1 min Cryo and 1 min Laser cycle treatments (20 min in total). Figure S19 (Supporting Information) shows the thermal mappings obtained by the single Cryo/Laser treatment cycle and **Figure** **5**A shows the temperature fluctuation curves. In particular, the Cryo treatment involved a series of freeze‐thaw cycles, each lasting for 30 s. After the initial application of liquid nitrogen‐soaked cotton, the temperature immediately decreased. The subsequent temperature rise in Figure 5A reflected the rapid thermal recovery to ≈11 °C before the next freeze‐thaw cycle was repeated. When 808 nm NIR laser was irradiated, the thermal recovery could reach ≈24 °C. There is a legitimate concern that cryotherapy can hinder the oxygen delivery due to the decreased blood flow, which has been proven to be normalized within the short periods with rewarming.^[^ ^59^ ^]^ The NIR laser irradiation generates a tissue heating (Figure 5A), which is assumed to enhance local blood flow to facilitate the supply of oxygen essential for the photodynamic therapy.

In vivo transdermal delivery of UCNPs@SiO_2_‐Ce6‐HA nanoplatforms was investigated by CLSM of cryo‐sectioned cancerous tissue sections derived from tumor regions after topical administration of PBS (Control), UCNPs@SiO_2_‐Ce6 and UCNPs@SiO_2_‐Ce6‐HA solutions for 30 min. CLSM showed the red fluorescence of UCNPs@SiO_2_‐Ce6‐HA nanoplatforms mainly in the entire inspected cancerous tissues (Figure 5B). In contrast, UCNPs@SiO_2_‐Ce6 exhibited poor skin penetration with strong fluorescence on the upper surface region of tumor tissues.

After treatment, mice were sacrificed and the digital photos of harvested tumors were taken as shown in Figure 5C, which clearly visualized the antitumor effect of the treatment groups. The tumor volumes and mice body weights were measured over a period of 14 days. Figure 5D shows no significant decrease in the body weights of mice over the treatment period, reflecting no significant toxicity from UCNPs@SiO_2_‐Ce6‐HA, Laser, and Cryo treatments. The administered nanoparticle dose appeared to cause negligible toxicity in vivo. As shown in Figure 5E, UCNPs@SiO_2_‐Ce6‐HA and Laser groups showed marginal tumor suppression compared to the control group. A slight delay in tumor growth was observed in Cryo and Cryo + UCNPs@SiO_2_‐Ce6‐HA groups, where tumor growth inhibition ratios were similar with ≈17%. Of note, cancer cells exhibited higher susceptibility to freezing injury compared to normal cells, as reported elsewhere.^[^ ^60^ ^]^ Notably, a statistically different inhibition of tumor growth was observed for mice treated in PDT groups. The only PDT (Laser + UCNPs@SiO_2_‐Ce6‐HA) treatment afforded the inhibition of tumor growth by more than 55% over 14 days. However, the most significant tumor volume regression of 79% was obtained in the Cryo‐PDT group (Cryo + Laser + UCNPs@SiO_2_‐Ce6‐HA). The successful Cryo‐PDT treatment might be attributed to the consecutive Cryo and Laser treatment cycles, which maintained the cooling of the tumor area for bright emission of UCNPs.

Finally, tumor sections were stained with hematoxylin and eosin (H&E) and exposed to terminal deoxynucleotidyl transferase‐mediated dUTP‐biotin nick end labeling (TUNEL) to examine tumor histology and cell apoptosis. Figure 5F shows the representative histological images of tumors after various treatments. Notably, tumor histological structures and cell survivals were not significantly changed in the cases of Control, UCNPs@SiO_2_‐Ce6‐HA, and Laser groups. Slight morphological changes and apoptosis were observed in Cryo and Cryo + UCNPs@SiO_2_‐Ce6‐HA groups. In consistent with the tumor growth, tumors treated with the Cryo‐PDT treatment group (Cryo + Laser + UCNPs@SiO_2_‐Ce6‐HA) showed the most severe tissue damage, and apparent cell apoptosis and necrosis on the H&E staining images. TUNEL assay also supported the antitumor effect with cell apoptosis exhibiting the strong green fluorescence signal in the major areas compared to the PDT treatment alone without cryo‐effect. Taken together, our proposed Cry‐PDT treatment successfully demonstrated the remarkable synergistic therapeutic efficacy against skin melanoma.

## 3 Conclusion

Inspired by the cryo‐responsive luminescence enhancement of UCNPs, we engineered the UCNPs@SiO_2_‐Ce6‐HA nanoplatform for the combined Cryo‐PDT treatment of skin melanoma. The low‐temperature environment enabled 2.45 times bright upconversion and significantly promoted a 3.15‐fold ^1^O_2_ production by the UCNPs@SiO_2_‐Ce6‐HA nanoplatform under 808 nm laser irradiation. Moreover, HA coating in the UCNPs@SiO_2_‐Ce6‐HA nanoplatform resulted in the effective transdermal delivery and the cancer cell‐targeting in the porcine skin and in vitro melanoma models. There was an impressive tumor growth inhibition of 79% in melanoma‐bearing mice by the combined cryotherapy and laser treatment, which surpassed the anticancer therapeutic effect of cryotherapy (17%) and PDT (55%) alone. In more general, our results uncover cryotherapy as a powerful tool to control the optical behavior of fascinating upconversion therapeutic systems. We expect that this study will bring about huge impact in the field of upconversion for biomedical applications and ultimately open a new avenue for the synergistic treatment of cryotherapy and PDT for melanoma skin cancers.

## 4 Experimental Section

### Materials

Reagents and solvents were acquired from commercial suppliers and used without any purification. Yttrium(III) acetate tetrahydrate (Y(CH_3_COO)_3_·4H_2_O, 99.9%), ytterbium(III) acetate hydrate, (Yb(CH_3_COO)_3_·nH_2_O, 99.9%), thulium(III) acetate hydrate (Tm(CH_3_COO)_3_·nH_2_O, 99.9%) and neodymium(III) acetate hydrate (Nd(CH_3_COO)_3_·nH_2_O, 99.9%), oleic acid (OA, tech. 90%) and 1‐octadecene (ODE, tech. 90%), ammonium fluoride (> 98%), cyclohexane (ACS grade), tetraethoxysilane (TEOS, 99.9%) and 1,3‐diphenylisobenzofuran (97%) were all supplied by Alfa Aesar Reagent Company. Methanol (99.9%), absolute ethanol (99.9%) and sodium hydroxide pellets (>97%), and sodium chloride were obtained from Daejung Chemicals & Metals Co., Ltd. Ammonium nitrate (≥98%), Cetyltrimethylammonium bromide (CTAB, ≥ 98%), (3‐aminopropyl)triethoxysilane (APTES, 99%), Deuterium oxide (D_2_O) and 4′,6‐diamidino‐2‐phenylindole (DAPI) were obtained from Sigma–Aldrich. Sodium hyaluronate (HA, MW = 100 kDa) was purchased from SNvia Co., Ltd. 1‐ethyl‐3‐(3‐dimethylaminopropyl) carbodiimide (EDC, > 98%), N‐hydroxysuccinimide (NHS, 98%) and diaminobutane (DAB) were purchased from Tokyo Chemical Industries Co., Ltd. Dulbecco's modified Eagle's medium (DMEM), fetal bovine serum (FBS), penicillin and phosphate‐buffered saline (PBS) were purchased from Invitrogen Co. Chlorine e6 was received from MedChem Express LLC. 2,7‐dichlorofluoroscin diacetate (DCFDA) was acquired from Cayman Chemical. The cell counting kit‐8 (CCK‐8) was obtained from Dojindo Molecular Technologies. Max‐View Live/Dead Staining Kit (Calcein‐AM/PI) was purchased from Biomax Co., Ltd. The mouse fibroblast (L929) and melanoma (B16F10) cell lines were acquired from Korean Cell Line Bank.

### Synthesis of UCNPs

UCNPs were synthesized through the Ostwald ripening strategy using the high‐temperature co‐precipitation method. In brief, the following lanthanide precursor solutions were prepared in 250 mL three‐necked round bottom flasks. Core solution‐A: Y(CH_3_COO)_3_ (0.2 m, 4 mL), Yb(CH_3_COO)_3_ (0.2 m, 1.9 mL), and Tm(CH_3_COO)_3_ (0.002 m, 0.5 mL); Shell solution‐B: Y(CH_3_COO)_3_ (0.2 m, 4.0 mL), Nd(CH_3_COO)_3_ (0.2 m, 1.0 mL). The flasks were heated to 110 °C for 10 min to evaporate water before adding a mixture of oleic acid (6.0 mL) and ODE (15.0 mL). Then, flasks were connected to the Schlenk line for degassing and nitrogen purging. The solution was kept at 150 °C for 30 min under gentle nitrogen flow to let lanthanide salts completely dissolve in the OA/ODE mixture. Next, 0.148 g NH_4_F and NaOH 0.1 g were dissolved in 9 mL methanol and injected into flask A, which was further heated to 110 °C to evaporate methanol and then degassed for 20 min. After the complete removal of the methanol, the mixture was heated at 300 °C under a nitrogen (N_2_) atmosphere for 1 h. Thereafter, preliminary precipitated, degassed, and heated to 150 °C (under N_2_) shell solution in flask B was injected into the solution in flask A at 300 °C, and the reaction further proceeded over 1, 1.5, and 2 h for size focusing purposes. The overpressure in the reactor system was prevented using a dual nitrogen balloon system. After cooling down to room temperature, the OA‐capped UCNPs were precipitated out by the addition of acetone and collected by centrifugation, washed with acetone several times, and finally redispersed in cyclohexane (20 mL) for further use.

### Synthesis of Mesoporous Silica‐Coated UCNPs

UCNPs were coated with mesoporous silica (SiO_2_) through the following procedure: first, 4 mL of UCNPs dispersion in cyclohexane was added into 40 mL DI water containing 0.32 g of CTAB under ultrasonication (Sonics VCX‐750 Vibra‐Cell Ultrasonic Liquid Processor, Sonics & Materials, Inc., Newton, CT, USA). Cyclohexane was further removed by gentle heating to 50 °C in the bath‐sonicator. Subsequently, 20 mL DI water was added into the solution and its pH was adjusted to 10.0 using 0.1 m NaOH solution, which was further homogenized using ultrasonication treatment for 30 min. Thereafter, TEOS was mixed well with absolute ethanol at a ratio of 1:4 by volume, respectively, and injected dropwise into the solution under continuous ultrasonication to prevent aggregation. Then, the solution was kept at 30 °C for 24 h under vigorous stirring. The synthesized UCNP@SiO_2_ were collected by centrifugation at 8000 rpm for 20 min and washed with ethanol several times. The CTAB surfactant was removed via the ion exchange method. The UCNP@SiO_2 _was transferred into the flask containing 0.3 g NH_4_NO_3_ dissolved in 40 mL ethanol. The flask was kept under ultrasound for 1 h at 50 °C. The CTAB extraction process was repeated two times. Next, the as‐obtained UCNPs@SiO_2_ were grafted with amine groups for further loading therapeutic components. First, UCNPs@SiO_2_ were re‐dispersed ethanol/water (95:5 by weight) solvent mixture with the subsequent addition of 100 µL APTES and stirred vigorously at 70 °C for 2 h under reflux. Finally, UCNPs@SiO_2_‐NH_2_ were isolated by centrifugation at 8000 rpm for 20 min and redispersed in 4 mL DI water for further use.

### Loading Ce6 Photosensitizer on the Mesoporous Silica‐Coated UCNPs

3 mg Ce6 was dispersed in 4 mL DI water and its carboxyl groups were activated via EDC/NHS chemistry. Then, 4 mL UCNPs@SiO_2_‐NH_2_ (10 mg) dispersion in DI water was added to the solution and sonicated for 10 min to form uniform dispersion. Then, the suspension was vigorously stirred at room temperature in the amber glass vial to avoid the interference of ambient light. After 24 h of reaction, UCNPs@SiO_2_‐Ce6 were precipitated out by centrifugation at 8000 rpm for 20 min. After removing the excess Ce6 by washing three times with DI water, the absorption spectrum of the collected supernatants was measured to quantify the unreacted Ce6. The loading capacity was calculated based on the calibration plot of the absorbance of the known Ce6 concentration solutions. Finally, the resultant UCNPs@SiO_2_‐Ce6 nanoplatform was freeze‐dried and stored at −20 °C in the dark.

### Synthesis of HA‐DAB

To synthesize HA‐DAB, HA with a MW of 100 kDa was dissolved in an ethanol/water (1:1 by volume) solvent mixture, and subsequently, DAB (40 molar ratios of HA repeating unit) was dissolved in the HA‐containing solution. Then, the EDC and NHS (2 molar ratio of HA) were introduced into the formed solution to activate the carboxyl groups of HA. The pH of the mixed solution was adjusted to 5.5 using 1 N HCl aqueous solution. The reaction was conducted under vigorous stirring at room temperature for 24 h, and the resulting HA‐DAB conjugate was dialyzed against 100 mm NaCl solution for 2 days, 25% ethanol solution for a day, and DI water for a day. The purified conjugate solution was freeze‐dried for 3 days. The effective degree of HA modification was analyzed by proton nuclear magnetic resonance (^1^H NMR) spectroscopy (AVANCE NEO 500, Bruker, Germany).

### Preparation of UCNPs@SiO_2_‐Ce6‐HA Nanoplatform

The as‐prepared HA‐DAB was conjugated on the surface of UCNPs@SiO_2_‐Ce6 (10 mg) via EDC/NHS coupling chemistry. The mixture was prepared in 10 mL of DI water and stirred for 24 h at room temperature in the amber glass vial. The resultant suspensions were dialyzed against DI water for 3 days in a dialysis bag with a molecular weight cut‐off of 7 kDa to remove unreacted molecules. The final freeze‐dried product was stored at −20 °C in the dark for future use.

### Physicochemical Characterization

The sample morphology images were acquired using an 80 kV transmission electron microscope (TEM, Talos, FEI, Lausanne, Switzerland). High‐angle annular dark‐field imaging coupled with energy dispersive spectroscopy (HAADF‐EDS) and selected area electron diffraction analyses were conducted using 200 kV TEMs, such as JEM‐2100F and JEM‐ARM200F (JEOL, Tokyo, Japan). Carbon contamination from TEM grids was removed using an ion cleaner JIC‐410 at 285 V for 5 min (JEOL, Tokyo, Japan). Crystallographic analysis was performed using Vesta software (Vesta Software Group, Wallingford, UK). The upconversion emission spectra were recorded using a spectrofluorometer (FL‐1039, HORIBA Scientific Co., Kyoto, Japan) using as the excitation light source an infrared diode laser system at 808 nm. The laser power density was adjusted by a power meter (843‐R, Newport Corp., Irvine, USA). The qualitative analysis of functional groups on the surface of nanoparticles was analyzed using a Spectrum Two Fourier Transform Infrared Spectrometer (Perkin Elmer Inc., Waltham, MA, USA). The crystal structure of UCNPs@SiO_2_ powder was analyzed using a PANalytical X'Pert Pro X‐ray diffractometer. UV/vis absorption spectra were recorded using a Scinco Mega‐800 spectrophotometer (Seoul, Korea). The lanthanide composition of UCNPs was analyzed using an Inductively Coupled Plasma Triple Quadrupole Mass Spectrometer (iCap‐TQ model, Thermo Fisher Scientific, Waltham, MA, USA). Dynamic light scattering and zeta potential measurements were conducted using a Malvern Pananalytical Zetasizer Nano ZS90 instrument (Malvern, UK). The particle size distribution analysis was conducted using ImageJ software (Rasband, W.S., ImageJ, U. S. National Institutes of Health, Bethesda, Maryland, USA).

### Extracellular Detection of Singlet Oxygen

One milliliter of UCNPs@SiO_2_‐Ce6‐HA (1 mg mL^−1^) aqueous solution was mixed with 1 mL of DPBF solution in ethanol in a 4 mL cuvette, then the sample was exposed to 808 nm laser with the power density of 0.5 W cm^−2^. The UV–vis absorption was measured ≈410 nm over 10 min (0, 2, 4, 6, 8, and 10 min). The cryo effect was investigated by keeping the cuvette at −80 °C for 1 min before laser irradiation. The temperature effect on the DPBF degradation rate was also conducted using UCNPs@SiO_2_‐Ce6 and blank DPBF solution.

### Cellular Uptake Study

The cellular uptake of samples was evaluated using L929 and B16F10 cell lines, which were acquired from the Korean Cell Line Bank (Seoul, Korea). B16F10 cells were seeded in 12‐well plates at a density of 1 × 10^5^ cells per well. Cells were cultured in a standard medium containing 10% FBS and 1% antibiotics for 1 day at 37 °C in a humidified atmosphere containing 5% CO_2_. Then, the cells were incubated with sample suspensions at a concentration of 500 µg mL^−1^ for 4 h. Before fixation with 4% paraformaldehyde, the cells were washed with PBS solution two times. Next, cells were stained with DAPI for 10 min before imaging with a confocal laser scanning microscope (ZEISS LSM 800, Oberkochen, Germany). With the aim to understand the role of HA receptor CD44 and LYVE‐1 during nanoparticle internalization, a competitive uptake inhibition was also performed on L929 and B16F10 cell lines with preliminary addition of excessive HA (100 kDa, 10 mg mL^−1^, 200 µL) for 30 min.

### Live/Dead Assay

B16F10 cells were seeded in a 24‐well plate at a density of 0.5 × 10^5^ cells per well. After cell culturing for 24 h, cells were incubated with nanoparticle suspensions at a concentration of 500 µg mL^−1^ for 4 h and then illuminated by an 808 nm laser at a power density of 0.5 W cm^−2^ for 10 min. Before imaging with a fluorescence microscope (EVOS M5000, Thermo Fisher Scientific, Waltham, MA, USA), the cells were incubated with Live/Dead cell assay solution for 10 min.

### Cell Viability Analysis by CCK Assay

The cytotoxicity of samples was assessed by CCK‐8 (tetrazolium salt) assays using L929 and B16F10 cell lines. The cells were seeded in 96‐well plates at a density of 1 × 10^4^ cells per well and cultured in a standard medium containing 10% FBS and 1% antibiotics for 1 day at 37 °C in a humidified atmosphere containing 5% CO_2_. The cells were then incubated with nanoparticle suspensions at different concentrations (10, 50, 100, 200, and 500 µg mL^−1^) for another day. Then, cell media was changed, and 10 µL of freshly prepared CCK‐8 assay was added to each well. After 4 h of incubation, the absorbance of CCK at 450 nm was measured using a microplate reader. The cell viability quantification was performed in accordance with the manufacturer's instructions.

### In Vitro ROS Generation Test

B16F10 cells were seeded in a 24‐well plate at a density of 0.5 × 10^5^ cells per well. After cell culturing for 24 h, cells were incubated with nanoparticle suspensions at a concentration of 500 µg mL^−1^ for 4 h and then illuminated by an 808 nm laser at a power density of 0.5 W cm^−2^ for 10 min. Before imaging with a fluorescence microscope, the cells were incubated with a DCFH‐DA probe for 30 min.

### In Vitro Photodynamic Therapy Treatment

B16F10 cells were seeded in a 96‐well plate at a density of 1 × 10^4^ cells per well for 24 h. Then, the cells were then incubated with nanoparticle suspensions at different concentrations (10, 50, 100, 200, and 500 µg mL^−1^). After 4 h incubation, wells were illuminated by an 808 nm laser at a power density of 0.5 W cm^−2^ for 10 min except for the control. Post‐laser incubation was conducted for 12 h, then cells were washed with PBS three times and incubated with CCK‐8 assay for relative cell viability quantification.

### Transdermal Delivery of UCNPs@SiO_2_‐Ce6‐HA Nanoplatform

The permeation ability of the UCNPs@SiO_2_‐Ce6‐HA nanoplatform across skin layers was evaluated using freshly excised porcine skin, which was obtained from the local slaughterhouse. UCNPs@SiO_2_‐Ce6 and UCNPs@SiO_2_‐Ce6‐HA nanoplatform were dispersed in PBS at a concentration of 500 µg mL^−1^ and topically applied on the surface of the porcine skin dermatomed to 0.5 × 0.5 cm using a freezing microtome (Leica, CM1860, Wetzlar, Germany). The transdermal delivery was assessed at 2, 4, 6, 12, and 24 h using a confocal laser scanning microscope (ZEISS LSM 800, Oberkochen, Germany). The fluorescence intensity was measured by ImageJ software.

### In Vivo Antitumor Effect Cryo‐Photodynamic Therapy Treatment

Female C57BL/6NcrlOri mice 5–6 weeks old were obtained from OrientBio Inc. (Seongnam, Korea). All the mice were cared for in the Pusan National University Animal Center under specific pathogen‐free conditions. This study protocol was reviewed and approved by the Institutional Animal Care and Use Committee (IACUC) of Pusan National University (Busan, Korea) in accordance with the National Institutes of Health guide for the care and use of experimental animals (Approval No.: PNU‐2023‐0264). Mice were randomly distributed to different groups before the treatment.

To evaluate the antitumor Cryo‐PDT effect, mice were subcutaneously inoculated with B16F10 cells at a density of 5 × 10^6^ to develop a tumor. Melanoma cells were suspended in the DMEM and injected into the right dorsal flank. After a week of inoculation tumor cells, the mice were randomly distributed into seven treatment groups (*n*  =  4, each group): group 1: Control applied with PBS solution (50 µL); group 2: UCNPs@SiO_2_‐Ce6‐HA (500 µg mL^−1^, 50 µL) without laser; group 3: Cryotherapy only (denoted as Cryo); group 4: 808 nm NIR laser treatment (denoted as Laser); group 5: Cryo and UCNPs@SiO_2_‐Ce6‐HA; group 6: Laser with UCNPs@SiO_2_‐Ce6‐HA (PDT); group 7: Cryo and Laser with UCNPs@SiO_2_‐Ce6‐HA (Cryo‐PDT). At the treatment stage, volume size reached an average size of 70 mm^3^. The tumor growth was monitored using the following equation: tumor volume =  *A* × *B* ^2^/2, where *A* and *B* are the maximum and minimum diameters of the tumor, respectively. The tumor surfaces of groups 2, 5, 6, and 7 received topical administration with the UCNPs@SiO_2_‐Ce6‐HA (500 µg mL^−1^, 50 µL) solution in PBS for 30 min. The Cryo treatment in groups 3 and 5 was conducted using a sterilized cotton pad pre‐dipped into the liquid nitrogen, which was then applied specifically to the tumor area in a series of repetitive freeze‐thaw cycles over a duration of 10 min. The Laser groups 4 and 6 were irradiated by 808 nm laser at a power density of 0.5 W cm^−2^ for 10 min. Notably, the combined Cryo‐PDT treatment in group 7 was conducted through the implementation of consecutive ten cycles, wherein each cycle was composed of 1 min Cryo (liquid nitrogen) and 1 min Laser treatment (0.5 W cm^−2^). Notably, the duration of the freeze‐thaw cycle in Cryo treatment was 30 s. The laser setup is illustrated in Figure S17 (Supporting Information). Thermal images were taken using FLIR E6390 infrared camera (Teledyne FLIR LLC, Wilsonville, USA). Posttreatment tumor sizes and mice body weights were measured and recorded over 14 days. The relative tumor volume was calculated by the ratio of the tumor volume to the initial tumor volume.

### Histological Analysis of Tumor Apoptosis

After 14 days of treatment, tumor tissues were harvested from all treatment groups, washed with PBS, and stored in 4% paraformaldehyde. Tumor tissues were dissected and embedded into the paraffin. To investigate the extent of tumor apoptosis, a hematoxylin and eosin staining kit (H&E, Tissue Protech.) and a terminal‐deoxynucleotidyl transferase mediated dUTP‐biotin nick end labeling (TUNEL) assay kit (DeadEnd Fluorometric TUNEL System, Promega) were used. The H&E and TUNEL staining procedures were conducted according to the manufacturers’ protocols.

### Statistical Analysis

Statistical analyses were conducted using unpaired, two‐tailed Student's *t*‐test or one‐way ANOVA using GraphPad Prism 5.0 (GraphPad Software, Inc., La Jolla, CA). Pairwise comparison of groups in terms of the tumor volume was performed using Tukey's test (*p* < 0.05) due to unequal group variance. The values of ^*^, *p* < 0.05; ^**^, *p* < 0.01; and ^***^, *p* < 0.001 were considered significant. All data are expressed as means ± standard deviation (SD) from at least three independent experiments.

## Conflict of Interest

The authors declare no conflict of interest.

## Author Contributions

A.M., H.E.C., and G.L. contributed equally to this work. S.K.H., K.S.K. conceptualized the idea for the study and performed supervision and funding acquisition; A.M. and H.E.C. contributed in synthesis of nanoplatform; A.M., G.L., J.M.P., J.H.K., H.B., J.P. contributed in characterization of nanoplatform; H.E.C. and D.W.H. performed In vitro and Ex vivo experiment; H.E.C., A.M., M.K., and S.B.L. performed animal experiment; A.M., H.E.C., G.L., S.K.H., and K.S.K. contributed in writing and original draft preparation; A.M., H.E.C., G.L., M.K, and S.B.L. performed visualization. All authors have read and agreed to be accountable for the content of this work.

## Supporting information

Supporting Information

## Data Availability

The data that support the findings of this study are available from the corresponding author upon reasonable request.
