# Supplementary material for: Cold‐Responsive Hyaluronated Upconversion Nanoplatform for Transdermal Cryo‐Photodynamic Cancer Therapy
Source: Adv Sci (Weinh). 2024 Mar 14;11(19):2306684. doi: 10.1002/advs.202306684 (PMC11109644; doi:10.1002/advs.202306684)
Supplement: Supplementary file 1 — Supporting Information [file ADVS-11-2306684-s001.pdf]

ADVANCED SCIENCE
Open Access

Supporting Information

for *Adv. Sci.*, DOI 10.1002/advs.202306684

Cold-Responsive Hyaluronated Upconversion Nanoplatform for Transdermal Cryo-Photodynamic Cancer Therapy

Anara Molkenova, Hye Eun Choi, Gibum Lee, Hayeon Baek, Mina Kwon, Su Bin Lee, Jeong-Min Park, Jae-Hyuk Kim, Dong-Wook Han, Jungwon Park, Sei Kwang Hahn and Ki Su Kim**

Supporting Information

Cold-Responsive Hyaluronated Upconversion Nanoplatform for Transdermal Cryo-Photodynamic Cancer Therapy

Anara Molkenova†, Hye Eun Choi†, Gibum Lee†, Hayeon Baek, Mina Kwon, Su Bin Lee, Jeong-Min Park, Jae-Hyuk Kim, Dong-Wook Han, Jungwon Park, Sei Kwang Hahn, Ki Su Kim**

† These authors contributed equally

A. Molkenova, H.E. Choi, M. Kwon, S. B. Lee, Prof. K. S. Kim

School of Chemical Engineering, Department of Organic Materials Science and Engineering, Institute for Advanced Organic Materials, Pusan National University, Busan 46241, Republic of Korea

E-mail: kisukim@pusan.ac.kr

G. Lee, Prof. S. K. Hahn

Department of Materials Science and Engineering, Pohang University of Science and Technology (POSTECH), Pohang 37673, Republic of Korea

E-mail: skhanb@postech.ac.kr

H. Baek, Prof. J. Park

School of Chemical and Biological Engineering, College of Engineering, Seoul National University, Seoul 08826, Republic of Korea

J. M. Park, Prof. J.-H. Kim

Department of Civil and Environmental Engineering, Pusan National University, Busan 46241, Republic of Korea

Prof. D.-W. Han

Department of Cogno-Mechatronics Engineering, BIO-IT Fusion Technology Research Institute, Pusan National University, Busan 46241, Republic of Korea

Table S1. Quantitative elemental composition of UCNPs based on the theoretical calculations and experimental data obtained from ICP-OES analysis.

Element [mol.%]	Y, mol.%	Yb, mol.%	Tm, mol.%	Nd, mol.%
Theoretical	80.3	9.5	0.1	10
Experimental	81.5	9.0	0.1	9.4

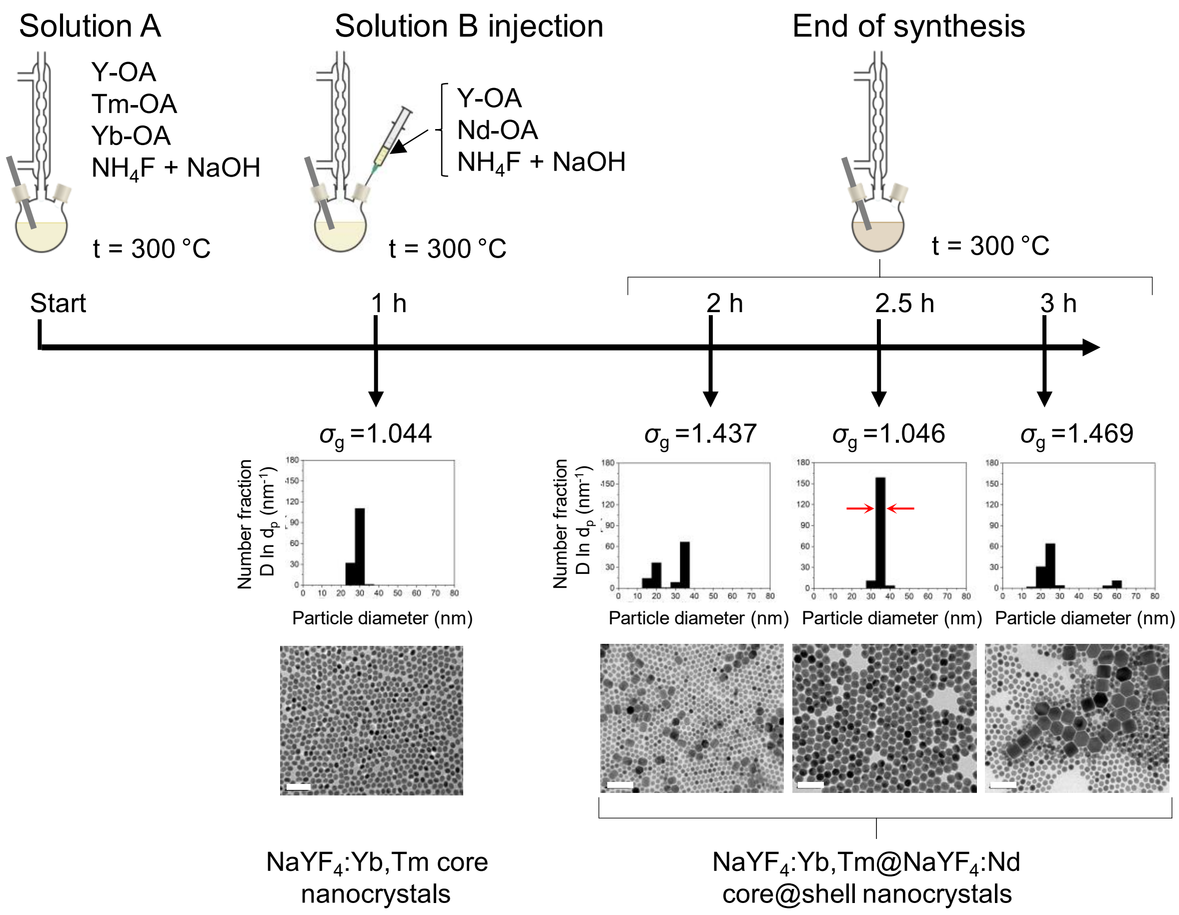

Figure S1. Schematic illustration of the reaction time optimization for size focusing of the core@shell upconversion nanocrystals prepared in a single step via Ostwald ripening strategy. Particle size distribution count number N = 300 and the scale bar in TEM images represent 100 nm. OA, oleate, σ_g, geometric standard deviation. The geometric mean diameter $d_{g,p}$, and geometric standard deviation σ_g were calculated from the equations as shown below:

$$\ln d_{g,p} = \frac{\sum_{i=1}^{N} \ln d_i}{N} \qquad (1)$$

$$\ln \sigma_g = \left[\frac{\sum_{i=1}^{N} \left(\ln d_i - \ln d_{g,p} \right)^2}{(N-1)} \right]^{1/2} \qquad (2)$$

respectively, where N is the total sample number and d_i is the measured particle size from TEM images.

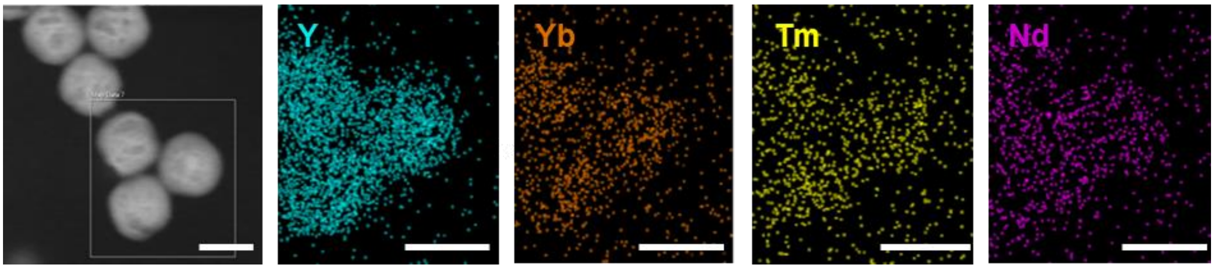

Figure S2. HAADF-STEM image and EDS elemental mapping images of UCNPs (scale bars: 25 nm).

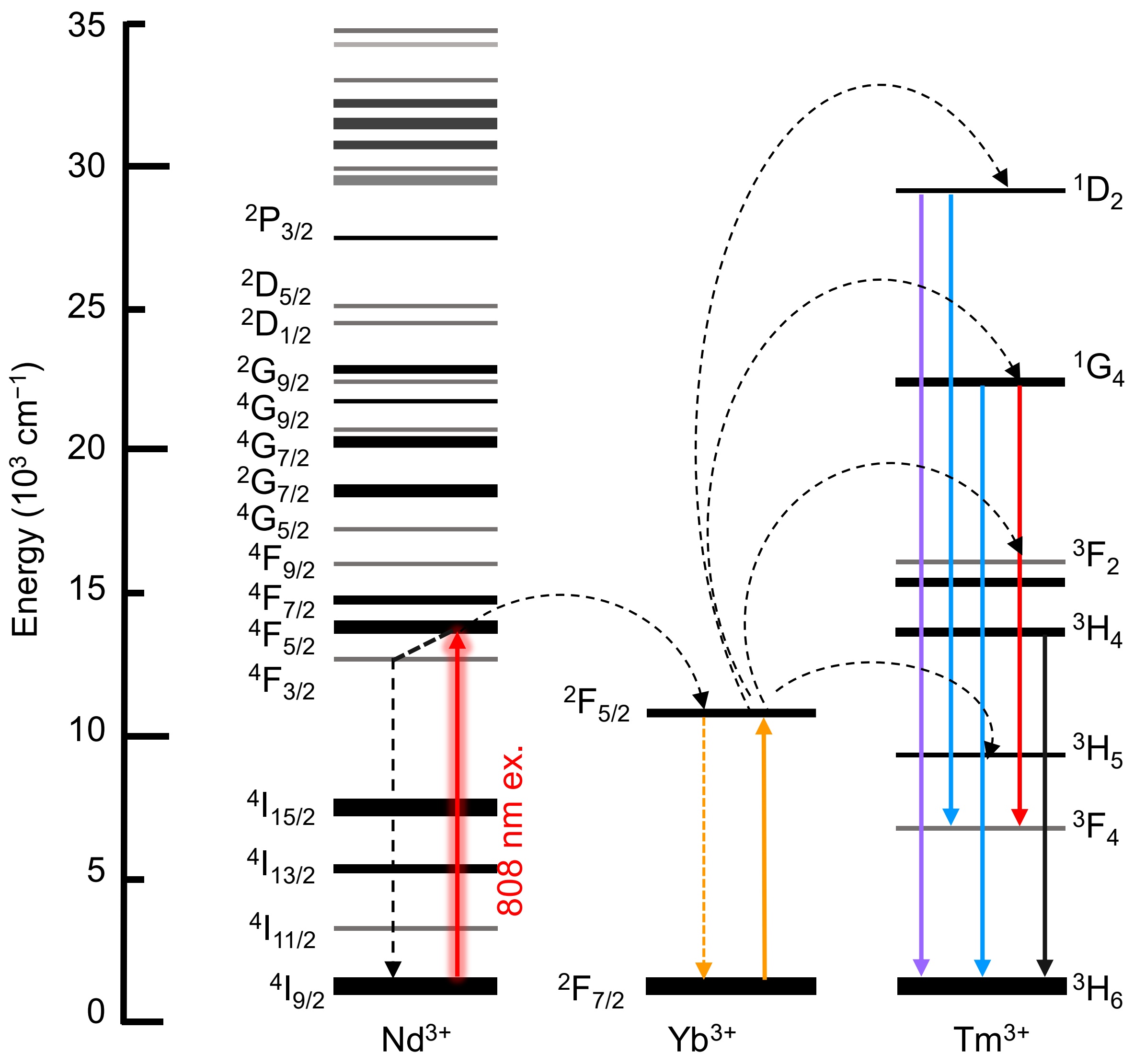

Figure S3. Schematic illustration of the upconversion process in $NaYF_4$:Yb,Tm@$NaYF_4$:Nd nanoparticles. Here Yb^{3+} serves as an energy mediator to transfer harvested photons from Nd^{3+} sensitizer to photoactivate Tm^{3+} emitter ion upon 808 nm excitation.

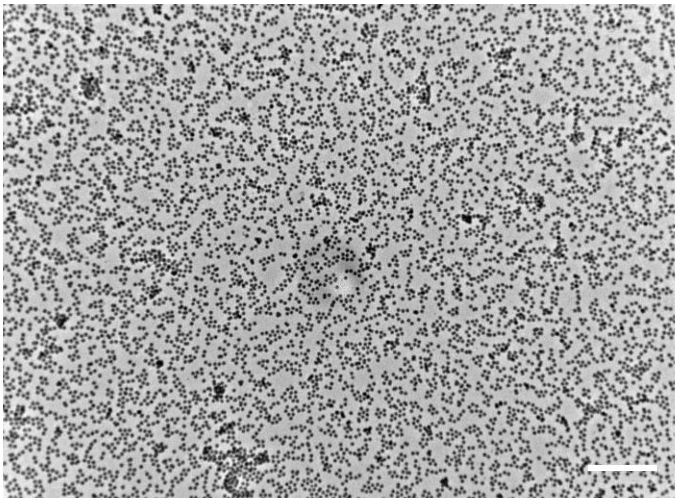

Figure S4. Low resolution TEM image of UCNPs@SiO_2 nanoparticles to validate the uniform coating without severe aggregation (scale bar: 1 μm).

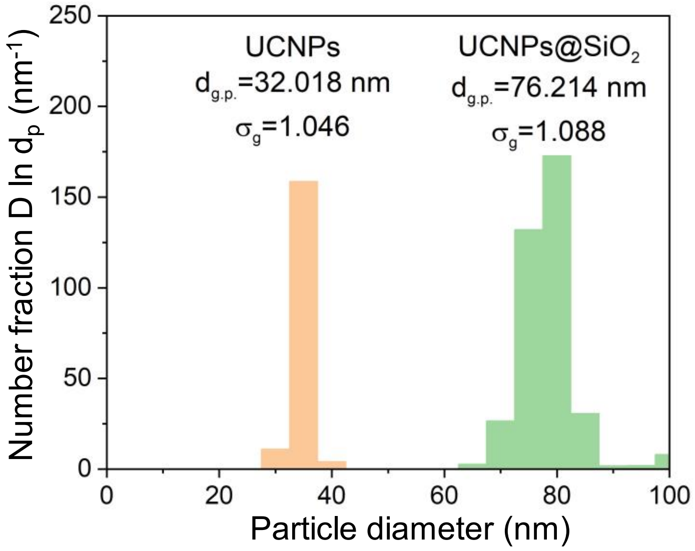

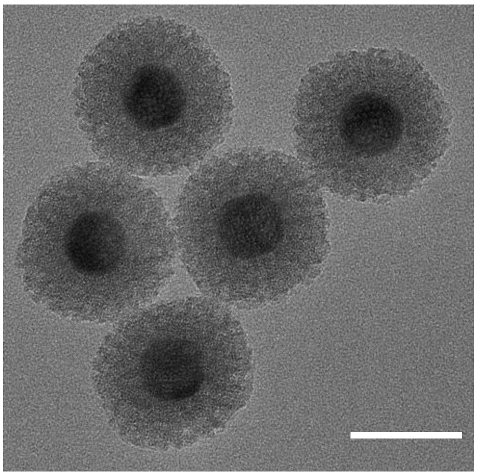

Figure S5. Particles size distributions of UCNPs and UCNPs@SiO_2 samples, where σ_g - geometric standard deviation; $d_{g,p}$ - geometric mean diameter. Count number N = 300. High resolution TEM image of UCNPs@SiO_2 (scale bar: 50 nm).

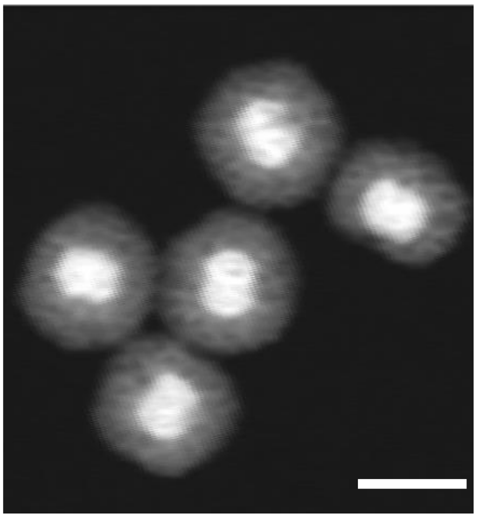

Figure S6. HAADF-STEM image of UCNPs@SiO_2 (scale bar: 25 nm).

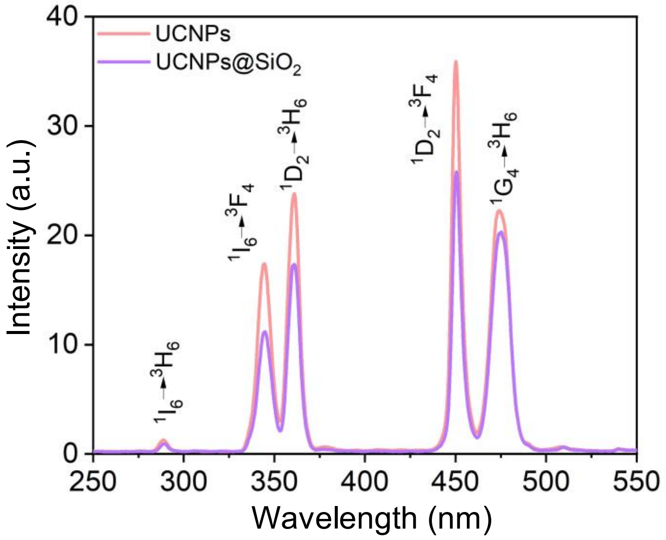

Figure S7. Upconversion emission profiles of UCNPs before and after silica shell encapsulation under 808 nm laser excitation at room temperature.

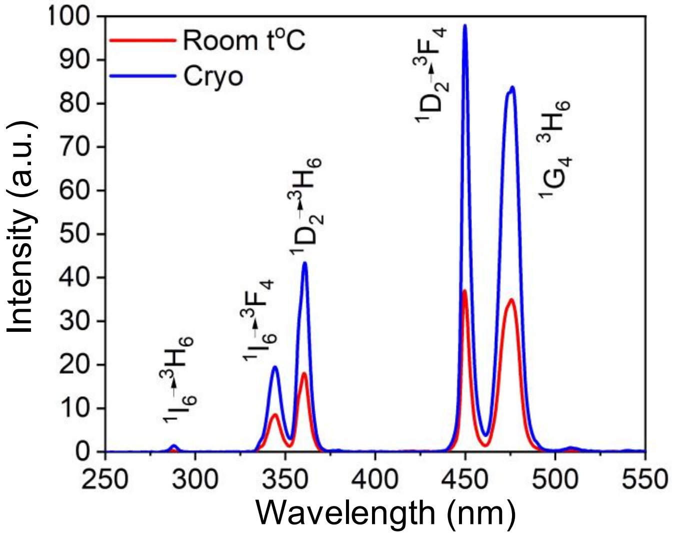

Figure S8. Upconversion emission profiles of UCNPs@SiO_2 sample at room temperature and cryo conditions.

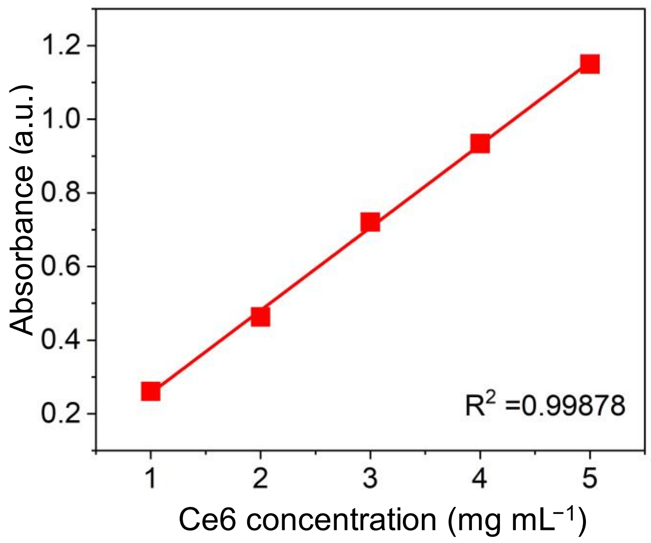

Figure S9. Calibration curve based on the absorption spectra of standard Ce6 solutions in dimethylformamide with different concentrations.

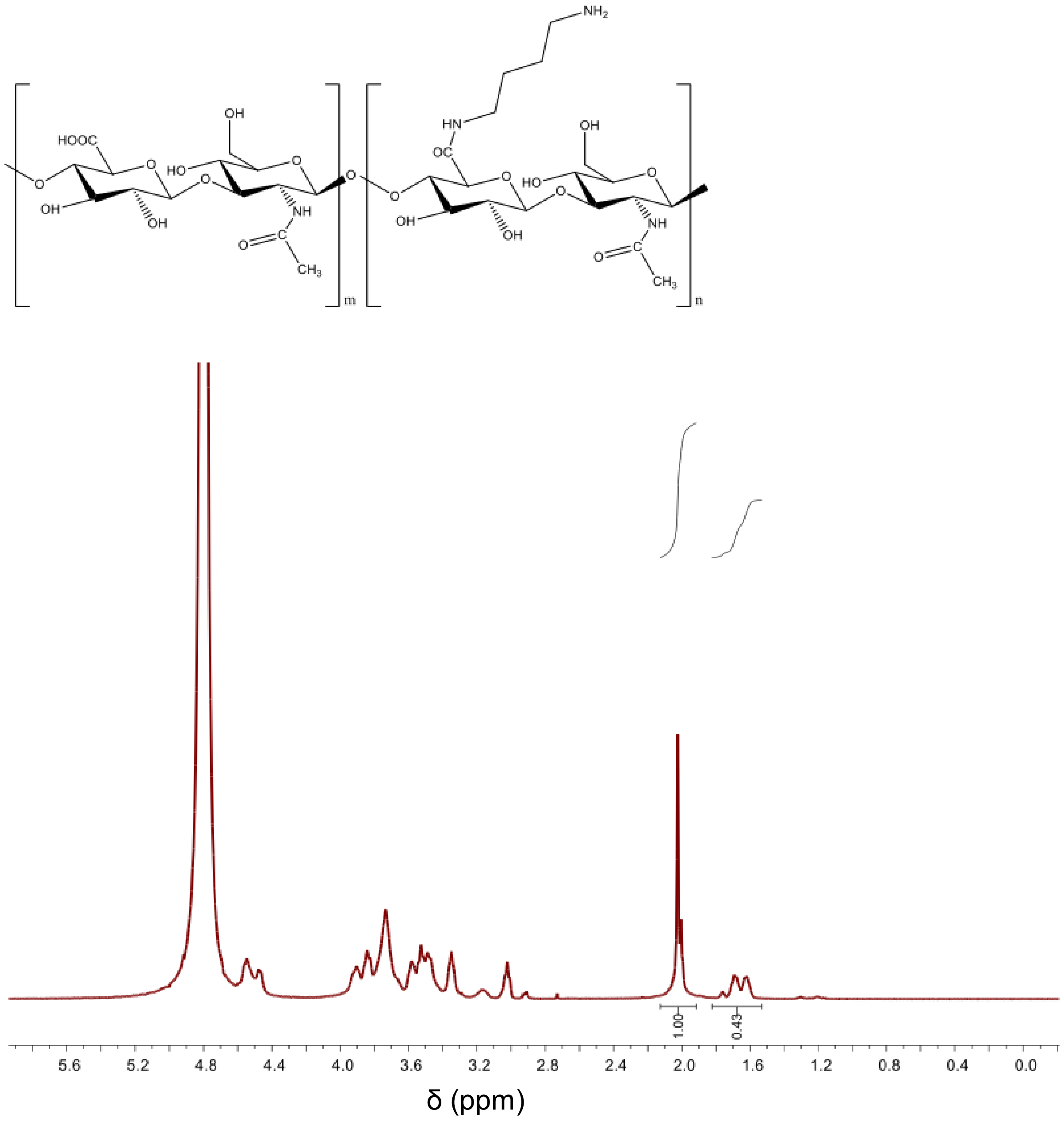

Figure S10. Chemical structure and ^{1}H NMR spectrum of the HA-DAB conjugate in D_2O.

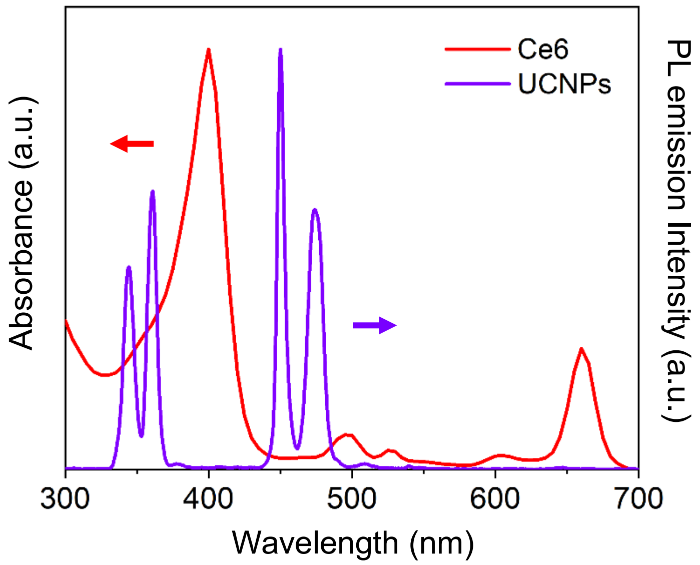

Figure S11. $NaYF_4$:Yb,Tm@$NaYF_4$:Nd core@shell UCNPs fluorescence excited at 808 nm and the absorbance of Ce6 photosensitizer.

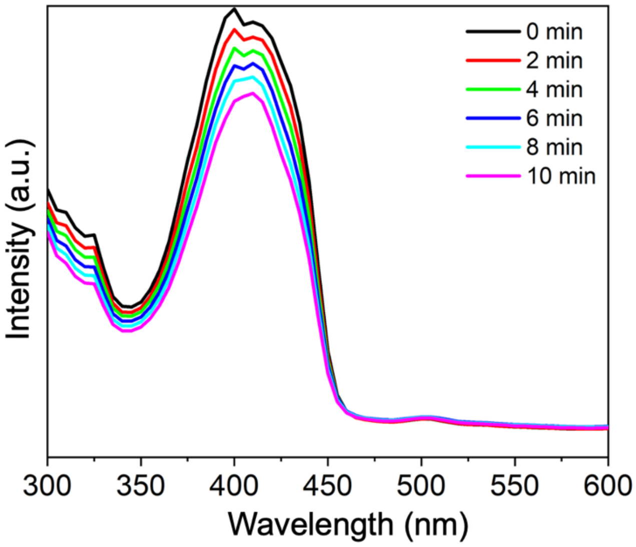

Figure S12. Room temperature singlet oxygen generation profile of UCNPs@SiO_2-Ce6-HA nanoplatforms under 808 nm laser irradiation using DPBF sensor.

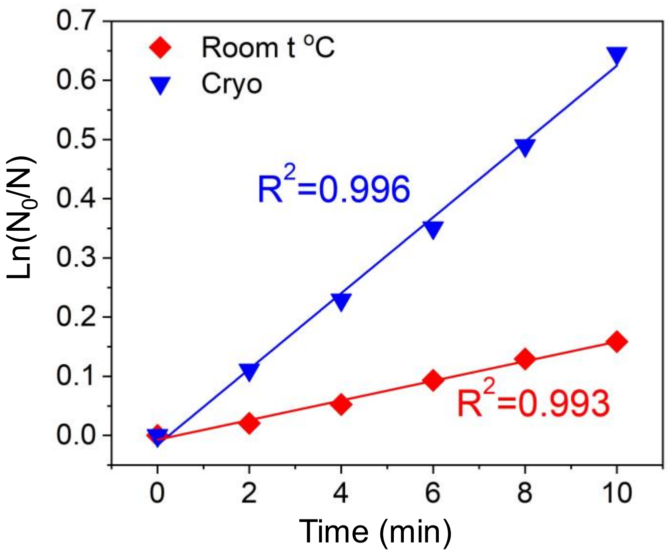

Figure S13. Comparison of DPBF photodegradation by UCNPs@SiO_2-Ce6 at cryo (blue, slope 0.0641, R^2=0.996) and room temperature (red, slope 0.0166, R^2=0.993) conditions.

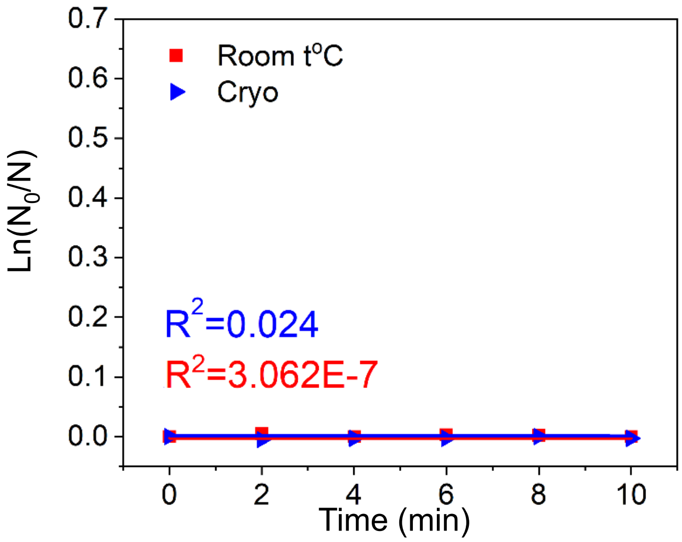

Figure S14. Blank DPBF sample degradation under 808 nm laser irradiation at room and cryo temperature conditions.

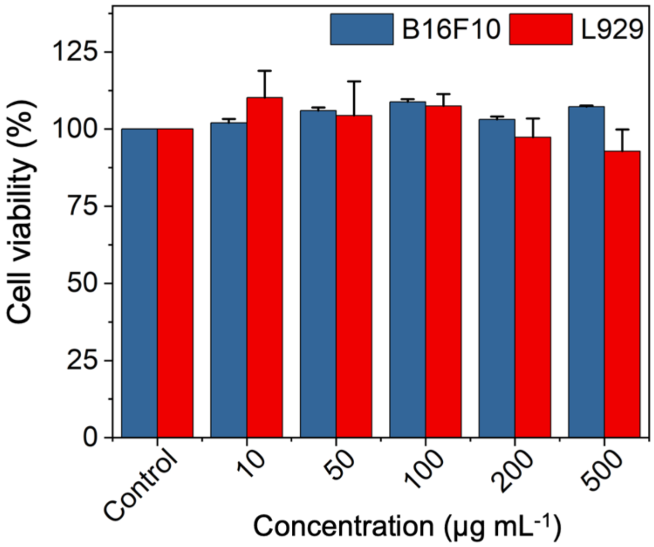

Figure S15. Relative cell viability of L929 and B16F10 cells, which were quantitatively assessed at 24 h post-incubation with varying concentrations of UCNPs@SiO_2-Ce6-HA nanoplatform (mean ± SD, n=3 experiments).

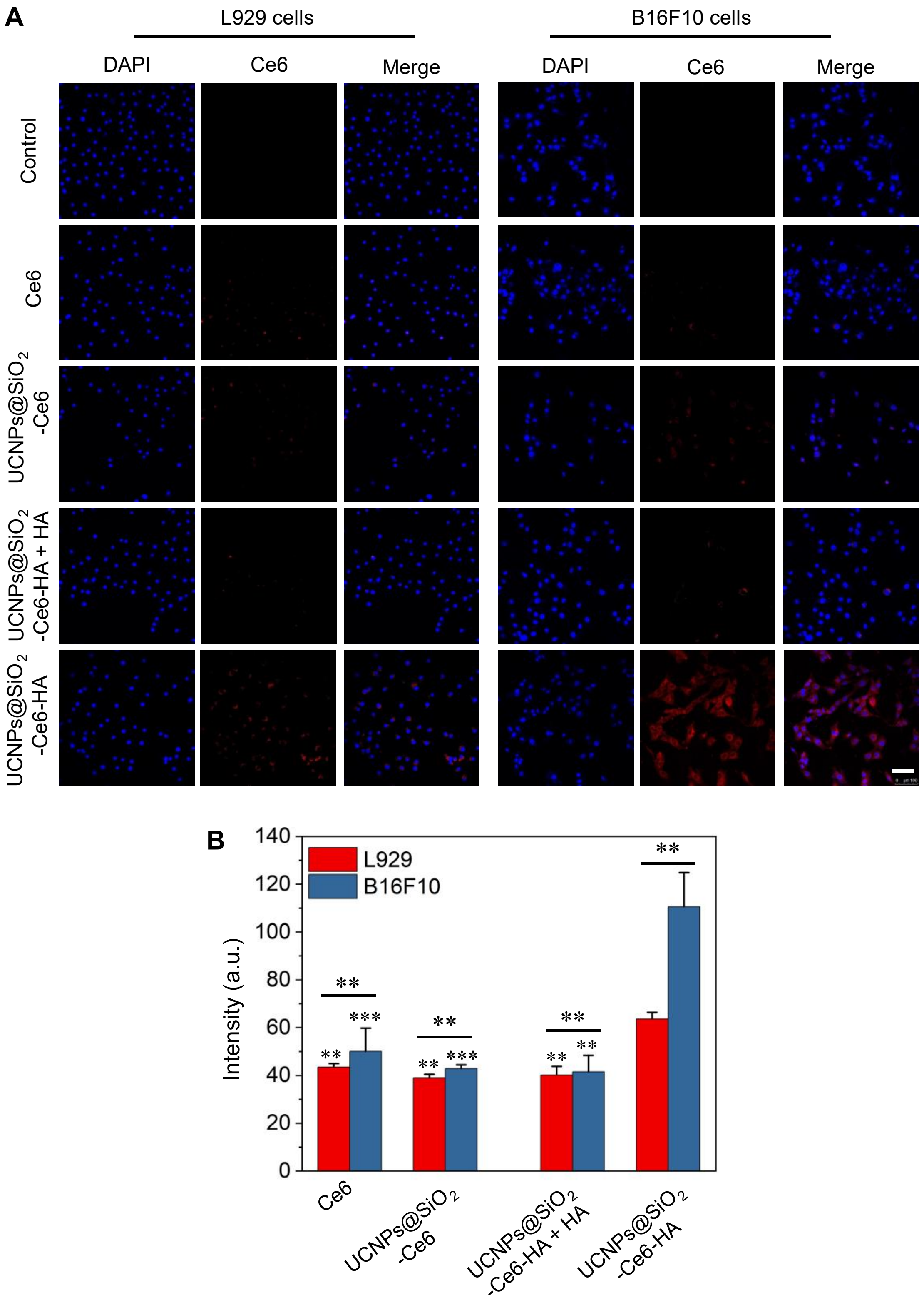

Figure S16. A) Confocal microscopic analysis of cellular uptake of UCNPs@SiO_2-Ce6-HA nanoplatform by B16F10 and L929 cells (scale bar: 100 μm). B) Quantitative fluorescence intensity results of (a) (n=3, **P<0.01, ***P<0.001 w.r.t. UCNP@SiO_2-Ce6-HA, and L929 versus B16F10 with incubation of UCNP@SiO_2-Ce6-HA).

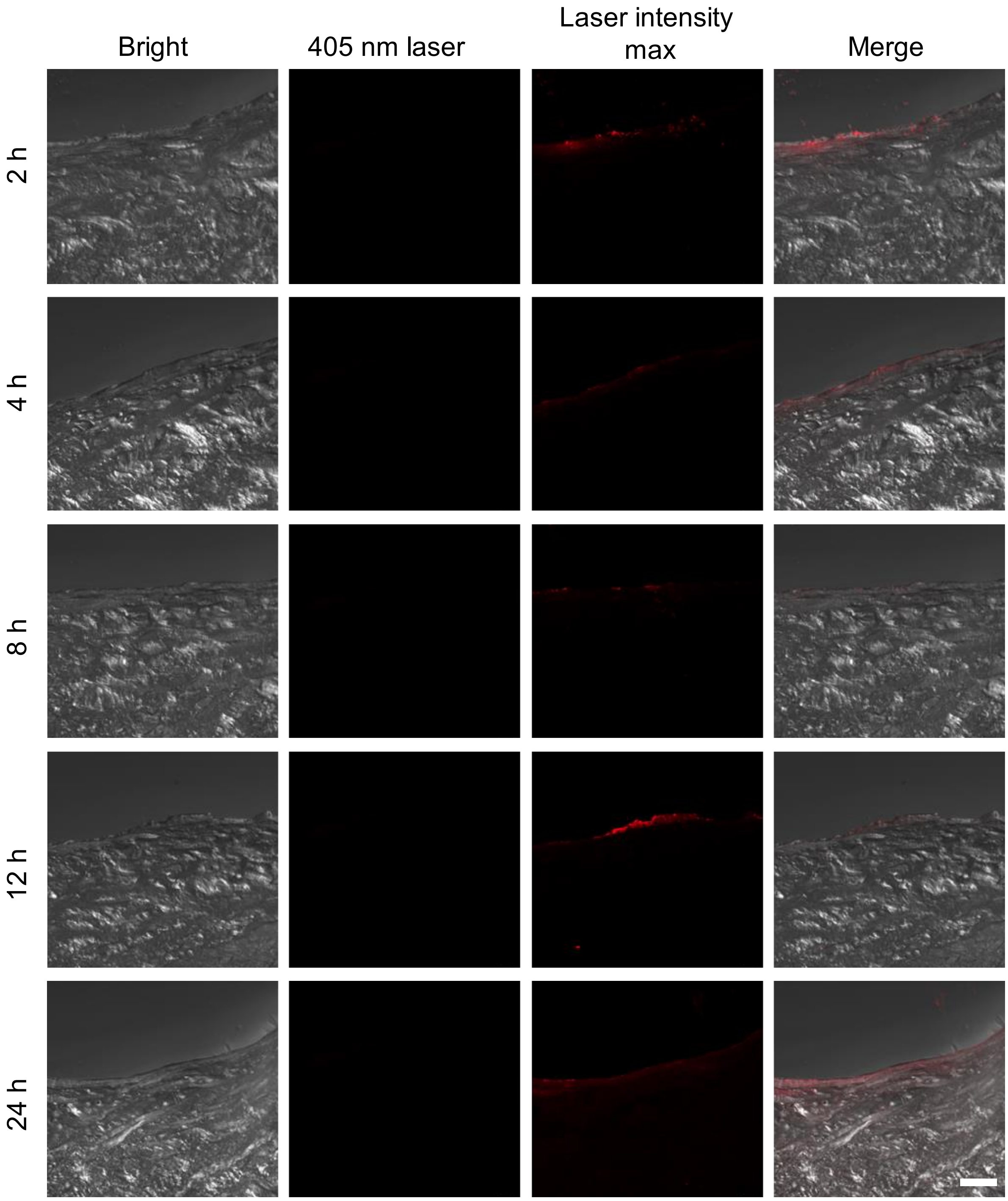

Figure S17. Confocal images of cryo-sectioned porcine skin harvested at 2 h, 4 h, 8 h, 12 h and 24 h post-topical administration of UCNPs@SiO_2-Ce6 nanocomposite particles. Images were obtained using maximum intensity of the CLSM 405 nm UV laser module with a maximum power of 5 mW (scale bar: 100 μm).

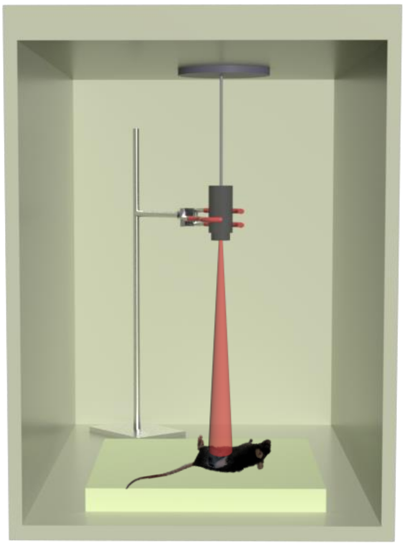

Figure S18. Scheme of the laser setup. The laser power density was adjusted by a power meter (843-R, Newport Corp., USA).

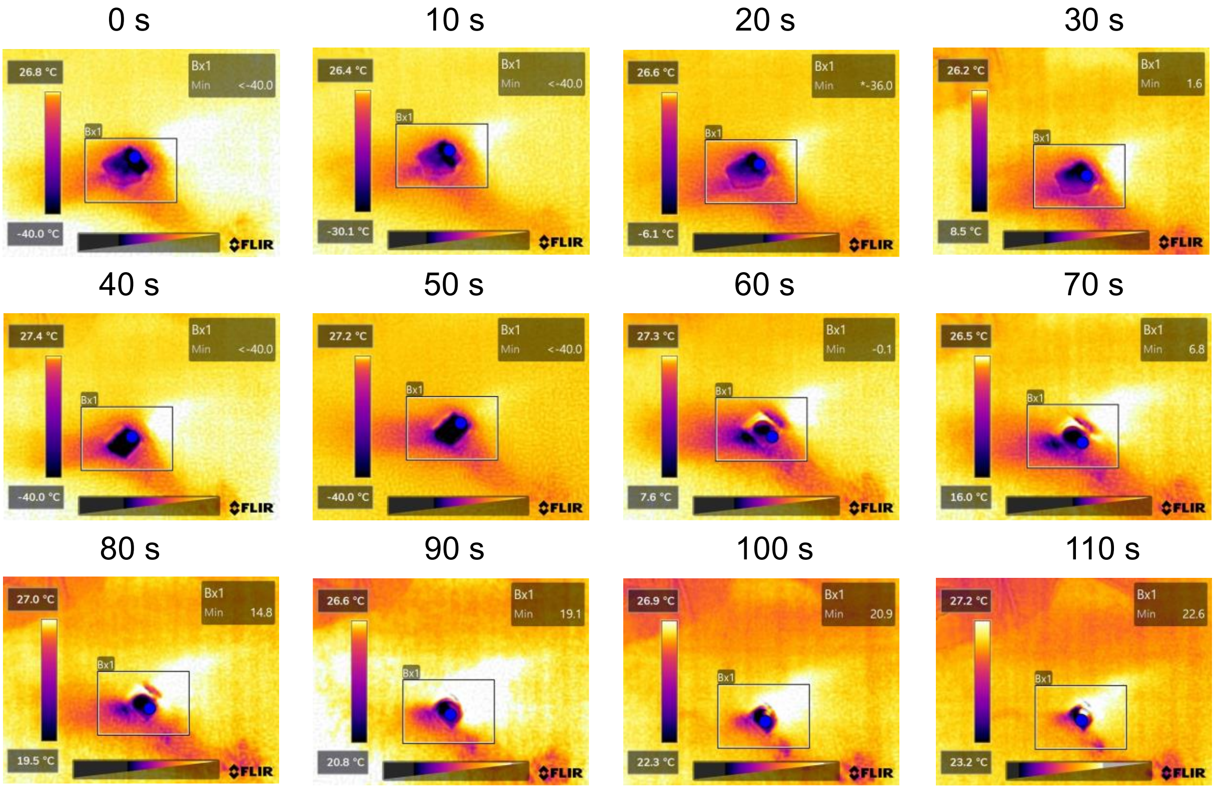

Figure S19. Thermal images captured during a Cryo-PDT cycle, which includes 1 min Cryo and 1 min Laser treatment. The duration of the freeze-thaw cycle was 30 sec. FLIR infra-red camera was used to take thermal images, which sensitivity of low temperatures was limited to -20°C.
